# Support for the efficient coding account of visual discomfort

**DOI:** 10.1017/S0952523824000051

**Published:** 2024-12-26

**Authors:** Louise O’Hare, Paul B. Hibbard

**Affiliations:** 1 NTU Psychology, Nottingham Trent University, Nottingham, UK; 2 Department of Psychology to Division of Psychology, University of Stirling, Stirling, UK

**Keywords:** SSVEP, edge orientation entropy, contrast, fractal dimension, spectral slope

## Abstract

Sparse coding theories suggest that the visual brain is optimized to encode natural visual stimuli to minimize metabolic cost. It is thought that images that do not have the same statistical properties as natural images are unable to be coded efficiently and result in visual discomfort. Conversely, artworks are thought to be even more efficiently processed compared to natural images and so are esthetically pleasing. This project investigated visual discomfort in uncomfortable images, natural scenes, and artworks using a combination of low-level image statistical analysis, mathematical modeling, and EEG measures. Results showed that the model response predicted discomfort judgments. Moreover, low-level image statistics including edge predictability predict discomfort judgments, whereas contrast information predicts the steady-state visually evoked potential responses. In conclusion, this study demonstrates that discomfort judgments for a wide set of images can be influenced by contrast and edge information, and can be predicted by our models of low-level vision, whilst neural responses are more defined by contrast-based metrics, when contrast is allowed to vary.

## Introduction

Visual processing is specialized for the efficient coding of the kinds of images that we typically encounter in our everyday environment (Barlow, 1961; Simoncelli & Olshausen, 2001). Efficiency is driven by principles such as sparse encoding by populations of neurons, whereby only a small proportion of neurons produce a strong response to any given input (e.g., Field, 1987, 1994, 1999). Sparseness is ensured by neurons having receptive fields and contrast gain responses that are tuned to the types of stimuli that are typical of the natural environment.

The theory of efficient coding is supported by analyses of the Fourier amplitude spectrum of images. The Fourier transform describes how an image can be decomposed into components of different spatial scales and orientations. Natural images have a characteristic amplitude spectrum, in which the amplitude (*A*) of components is close to inversely proportional to spatial frequency (*f*), producing an approximately *A* ∝ *f^k^* relationship, with *k* taking a value of around −1 (Burton & Moorhead, 1987; Field, 1987; Tolhurst et al., 1992). The contrast sensitivity function, which describes how our sensitivity to visual stimuli varies with frequency, shows a peak sensitivity to midrange frequencies. This spatial frequency tuning has been explained as an efficient encoding of stimuli with the 1/*f* amplitude spectrum that is typical of natural images (Atick & Redlich, 1992).

Deviations from these statistical properties of natural images have been associated with visual discomfort or visual stress (Juricevic et al., 2010). Images that create discomfort in viewers tend to have excess contrast at midrange spatial frequencies, to which the visual system is especially sensitive (Fernandez & Wilkins, 2008; Juricevic et al., 2010; O’Hare & Hibbard, 2011; Wilkins et al., 1984). Hibbard and O’Hare (2015) showed how these results can be related to the efficient encoding of images by the visual cortex. Using a simple feed-forward model of receptive fields in the primary visual cortex, we showed that uncomfortable stimuli will tend to produce large and non-sparse neural responses. Moreover, Penacchio and Wilkins (2015) have shown the degree to which the amplitude spectra of images deviate from those of typical natural images is a strong predictor of visual discomfort. Fourier spectral slope analysis provides an explanation of the visual discomfort created by some architectural (Le et al., 2017) and typographic (Wilkins et al., 2007, 2020) designs. An excess of the type of visual content to which the visual system responds most strongly has been hypothesized to create discomfort through excessively large neural responses (Hibbard & O’Hare, 2015; Wilkins & Hibbard, 2014). Large responses to uncomfortable stimuli have been shown using visually evoked potentials (O’Hare et al., 2015; O’Hare, 2017a), functional near infrared spectroscopy (Le et al., 2017; Shi et al., 2022), and fMRI (Huang et al., 2011).

The current study tested whether visual discomfort results from excessive responses to stimuli that are not well-matched to the statistical properties of images for which the visual system is optimized (Hibbard & O’Hare, 2015; O’Hare & Goodwin, 2018; O’Hare et al., 2021; Wilkins & Hibbard, 2014). Previous computational modeling work has quantified how the visual system will respond to uncomfortable images (Hibbard & O’Hare, 2015; Penacchio et al., 2023; Penacchio & Wilkins, 2015). Other studies have measured behavioral (Bies et al., 2016; Fernandez & Wilkins, 2008; Juricevic et al., 2010; O’Hare & Hibbard, 2011; Spehar et al., 2003; Taylor et al., 1999) or neural (O’Hare & Goodwin, 2018; O’Hare et al., 2021) responses to uncomfortable stimuli, artworks, and natural images. However, there is not yet an integrative study that combines computational modeling, image statistics, subjective judgments, and neural responses to the same stimuli. This combined approach is essential to determine whether our computational and statistical models can account for both the special properties of artworks as visual stimuli, and the relationship between neural responses and visual discomfort.

We measured the low-level statistical properties of artworks, natural images, and uncomfortable stimuli (Fourier amplitude spectrum, fractal dimension, edge orientation anisotropy, and physical and effective contrast (following image filtering to take account of the contrast sensitivity function)). We also calculated the expected neural response to each stimulus using a simple feed-forward model of the primary visual cortex (Hibbard & O’Hare, 2015). Each stimulus was rated for discomfort so that we could understand how this varied across stimulus categories, and the degree to which it could be predicted from the statistical properties of the images, and the predicted neural response. Finally, we also measured these responses directly using steady-state visually evoked potentials (SSVEPs). In this way, we combined computational, psychophysical, and physiological measures, and used a broad range of stimuli that are expected to produce both high and low levels of visual discomfort. This allowed us to test the prediction that discomfort is related to a high level of neural activity that is driven by the statistical properties of uncomfortable stimuli.

Natural images, artworks, and uncomfortable images were included in the study to provide a broad range of discomfort levels. Natural images provided a baseline category, for which visual encoding is hypothesized to be optimized (Barlow, 1961; Simoncelli & Olshausen, 2001). Sinusoidal gratings and bandpass-filtered noise stimuli were included since their statistical properties vary from those of natural images in ways that have been associated with visual discomfort (Fernandez & Wilkins, 2008; Juricevic et al., 2010; O’Hare et al., 2015; O’Hare & Hibbard, 2011; Wilkins et al., 1984). We used grayscale images since the role of luminance statistics in discomfort and visual encoding is well established (e.g., Fernandez & Wilkins, 2008; Juricevic et al., 2010; O’Hare et al., 2015; O’Hare & Hibbard, 2011; Wilkins et al., 1984). While the current study did not assess the chromatic properties of images, these are also known to contribute to visual discomfort (Haigh et al., 2013; Juricevic et al., 2010; Penacchio et al., 2021). Similarly, this focus on low-level image properties also does not address the roles of perceptually high-level and semantic factors in visual discomfort.

In contrast to highly artificial, uncomfortable images, artworks have statistical properties that may be expected to be related to higher levels of viewing comfort than natural images. Artworks tend to have a slope closer to *k* = −1 than mundane images, which has been taken as evidence that artists are attempting to create images with an amplitude spectrum closer to optimal for the visual system (Redies et al., 2007; Redies et al., 2008; Graham and Field, 2007a; Graham & Field, 2008; Mather, 2013, p. 149; Koch et al., 2010). This means that these images might be considered idealized stimuli for sensory encoding in comparison with natural images, and will therefore elicit low levels of discomfort. In contrast, we also included two artworks by Bridget Riley, whose artworks have been associated with visual discomfort (Dodgson, 2012; Wilkins et al., 1984).

We have several research questions. Firstly we assessed whether discomfort judgments would be predicted by models of early visual processing, and by the size of neural responses measured at the scalp. Secondly, we assessed whether images creating higher neural responses measured at the scalp would be judged as more uncomfortable, and secondary to that we thus predicted that the smallest SSVEP responses would be elicited by artworks, and the largest responses by grating and bandpass filtered noise. Thirdly, we also predicted the largest responses and greatest levels of discomfort would be seen for stimuli with midrange spatial frequencies in stimuli where spatial frequency was systematically varied. The exceptions to these predictions were the two artworks by Riley, which we predicted would elicit relatively large neural responses, and high levels of visual discomfort. Finally, we predicted that low-level image statistics (spectral slope, fractal dimension, measures of contrast, edge orientation entropy) would predict discomfort judgments and neural responses. In addition, and in contrast with previous literature that tends to look at individual image statistics, we will investigate dimension reduction, as many of the image statistics are highly interrelated.

## Materials and methods

### Stimuli

Artwork stimuli were taken from www.prometheus-bildarchiv.de following work by Wallraven et al. (2008, 2009). The categories of artwork chosen followed those used in these papers. The reasoning for including several genres was to sample broadly, rather than to consider genre itself systematically. Works by Bridget Riley were included as a distinct category due to the known association between her artworks and visual discomfort (Dodgson, 2012; Wilkins et al., 1984). For the EEG experiment, a subset consisting of two examples from each of the categories was chosen. The full set of images used in the computational model was 514 images. It would not be feasible for a participant to view such a large number of stimuli. A more limited sample of 48 images was chosen for the EEG experiment compared to the model as the human participant is limited in terms of attention and fatigue. Testing over more than one session would introduce additional variability in terms of different levels of noise between sessions for each observer in terms of time of day, caffeine consumption, electrode placement and so on as well as possible attrition that it was felt would be better avoided. The complete list of artworks from each genre presented in the EEG experiment can be seen in Table 1.

**Table 1.** List of artworks included in the EEG experiment

Genre	Artist	Artwork
Baroque	Caravaggio	Amor Vincit Omnia
Baroque	Rubens	The Consequences of War
Classicism	Ingres	Raphael and La Fornarina
Classicism	Jacques	The Oath of the Horatii
Expressionism	Kandinsky	Zuer Ecken
Expressionism	Munch	Two Young Women in Red and White
Impressionism	Monet	Summer
Impressionism	Renoir	Chestnut Tree in Bloom
Realism	Courbet	Portrait of Bruyas
Realism	Millet	Roman Landscape with Finding of Moses
Renaissance	Da Vinci	The Adoration of the Magi
Renaissance	Raffael	Madonna Colonna
Rococo	Watteau	The Dance
Rococo	Boucher	Landscape with Farmhouse
Romantic	Turner	The Fall of an Avalanche in the Grisons
Romantic	Goya	The Sleep of Reason Produces Monsters
Surrealism	Dali	Portrait of Frau Isabel Styler-Tas
Surrealism	Magrite	L’Idee Fixe
Suprematism	Malevich	Taking in the Rye
Suprematism	Malevich	Bureau and Room
Modernism	Mondrian	Broadway Boogie Woogie
Modernism	Mondrian	Chequerboard Dark Colors
Op-Art	Riley	Fall
Op-Art	Riley	Blaze

It is important to note that all artwork images were gray-scaled for use in the current study using the MATLAB rgb2gray function. This is due to comparability with the natural images and artificial images that were both in grayscale, and the additional complexity of estimating low-level image statistics for color stimuli. Whilst this is possible, it would not be able to directly compare the image statistics for the different image categories.

Natural images were 200 images taken from the van Hateren image database (van Hateren & van der Schaaf, 1998). For the EEG experiment, a subset of natural images was chosen at random, corresponding to image numbers: 13, 17, 28, 34, 41, 47, 84, 94, 103, 106, 129, 146, 161, 179. Filtered noise patterns with different spatial frequency content “bump” stimuli were created following Fernandez and Wilkins (2008). As these stimuli are created following this article, we also use the terminology of Fernandez and Wilkins (2008). These consist of filtered noise patterns created using a raised radial cosine function (see equation (1)).

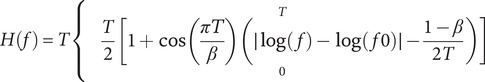



(1)

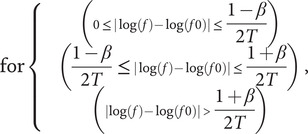

where T is 0.9, β is 0.5, *f* is the spatial frequency, and f0 is the center frequency of the function, defining the peak of the “bump.” For the model, the center frequencies were 0.1875, 0.375, 0.75 1.5, 3, 6, and 12 cycles/degree. For the EEG experiment, the center frequencies were 0.75, 1.5, 3, 6, and 9 cycles/degree. Finally, vertical sinusoidal gratings of spatial frequencies were included, for the model, these were 0.1875, 0.375, 0.75, 1.5, 3, 6, and 12 cycles/degree, for the EEG experiment, this was a shorter list of 0.75, 1.5, 3, 6, and 9 cycles/degree. The lower spatial frequencies were truncated as the mid-range spatial frequencies have been shown in previous work to be the most uncomfortable (O’Hare & Hibbard, 2011).

Examples of the artificial stimuli can be seen in the Open Science materials accompanying this article, hosted at the Open Science Framework: https://osf.io/zcfuw/. The images of the artworks are not able to be included in the repository as these are hosted elsewhere: www.prometheus-bildarchiv.de. Samples of natural images from the van Hateren database are likewise not reproduced as these are publicly available through the van Hateren database: https://github.com/hunse/vanhateren.

Importantly, images were not matched for physical contrast as this has been done in previous work (e.g., O’Hare et al., 2021) and one of the aims of the study was to allow contrast to vary to be able to account for its contribution to discomfort.

### Computational model

Following Hibbard and O’Hare (2015), a model of the visual system was made of 500 model cells with a range of spatial frequency and orientation tuning taken from biologically plausible distributions. Model cells were created using log Gabor functions, using the DoLogGabor.m function (Goffaux & Dakin, 2010). We used distributions of cell properties based on physiological data for spatial frequency (Devalois et al., 1982) orientation (Li et al., 2003), and phase (Ringach, 2002). We assumed an orientation bandwidth of 16–17 degrees, also based on physiological data (Ringach, 2002). Images were filtered using the 500 model cells, and the total model output, as well as model response kurtosis, was estimated for each image. Detailed model responses for each image category can be seen in the Supplementary Material.

### Image statistics

Images were analyzed for their low-level statistical properties, including spectral slope, fractal dimension, CSF-filtered contrast, and edge orientation entropy.

Spectral slope was estimated by taking the log Fourier transform of the image and plotting the amplitude against log spatial frequency and fitting a first-order polynomial. The resulting slope value was taken as an estimation of spectral slope.

Fractal dimension was estimated by box-counting (Li et al., 2009). The image is first posterized into bounded regions, using a cut-off of 128, half the maximum possible value of the pixels of the image. All images were first grayscaled using the rgb2gray function in MATLAB, and so in this case, although the original artworks would have been in color, all stimuli in the current study were converted to grayscale first. The box sizes are defined in powers of 2, up to a maximum limited by the number of pixels in the longest dimension of the image. This limit is the smallest possible integer, that 2 can be raised to, that is larger than the maximum dimension (in pixels) of the image. For example, considering an image of 300 pixels by 300 pixels, the smallest possible integer *x* that satisfies 2^*x* would be 9. The image is zero-padded to the maximum box size. The number of boxes needed to cover the non-zero elements of the posterized image is counted for each of the box sizes. This results in a function of the number of boxes against box size. The local slope of the function of the number of boxes against box size can be estimated using the following equation:
(2)

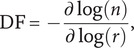

where *n* is the number of boxes and *r* is the box size in pixels. The gradient of this slope is constant for a series of box sizes, then this can be the estimate of the fractal dimension.

CSF-filtered contrast was calculated by applying the contrast sensitivity function to the image following the equation of Mannos and Sakrison (1974).
(3)

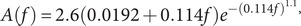

where *f* is the spatial frequency of the image in cycles per degree, up to a limit of 60 cycles per degree.

**Figure 1.**
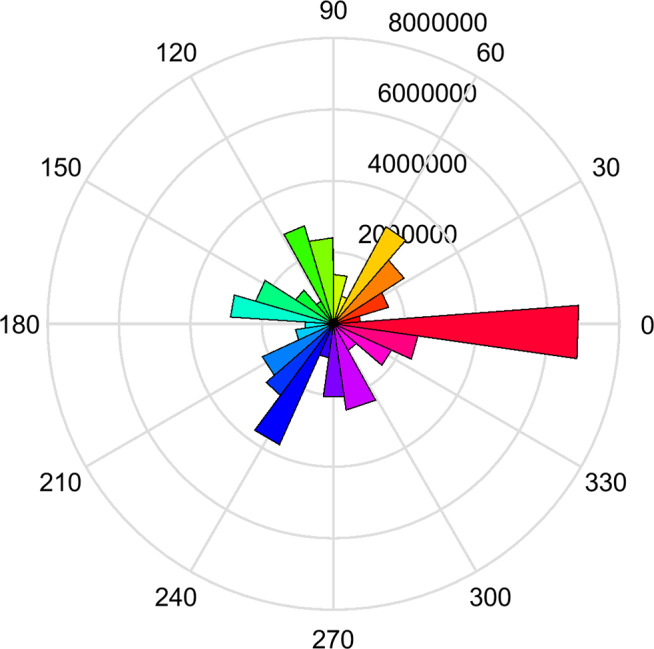
Circular plot showing distribution of the edge information (orientation differences) contained in one example natural image (the final in the set). Orientation is defined across the full range of 0–360°, such that a rotation through 180° produces a reversal in the contrast polarity of the edge.

Edge orientation entropy was estimated using the method of Redies et al. (2017). First, each image was converted to greyscale using the “rgb2gray” function in MATLAB. Then images were scaled down to a maximum size of 340 × 340 pixels using the function “imresize” for ease of analysis. A set of 24 Gabor filters was used to determine the edges of each image for a range of orientations. The edges of each image were determined using the following Gabor function:
(4)

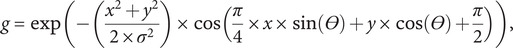

where *x* and *y* are the image pixels, *σ* is 1.669 (following Redies et al., 2017), and 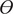 varied from 0 to π in 24 steps. Images were convolved with the filter array to identify the edges. The responses to the 15 pixels at the edges of the images were discarded to limit border effects. Each edge was then compared pairwise to every other edge identified in the image. The highest response of the filter array determined the overall orientation of the edge. Only the highest 10,000 edge responses were included in the analysis, following Redies et al. (2017). The intensities of the two edges were multiplied together (the edge-pair intensity product). Histograms of these products were created with each orientation as bins. The bins of the histograms of edge-pair intensity products were determined by the Euclidean distance between the two edges (*d*) and the angle between the two edges (alpha). There were 500 bins for *d* and 48 bins for α. For each bin defined by *d* and α, histograms of the angles were normalized, and the probability of each edge occurring was estimated. The maximum possible for each section is 1/24 if there is an even spread of orientations throughout the image. The Shannon entropy is estimated using the following equation:
(5)

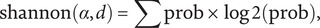

where α is the angle between the two edges bin, *d* is the Euclidean distance bin, and *prob* is the probability of the edge occurring compared to the even spread of orientations occurring in the image. A circular plot showing a histogram of the orientation differences for one example natural image can be seen in Fig. 1. Shannon entropy was averaged over α and *d* for each image.

Details of the image statistics including the results over image category can be seen in the Supplementary Material.

### Apparatus

Stimuli were displayed using an Asus Prime computer with an Intel i7 core and NVidia GForce graphics card, using an Ubuntu 14 operating system. The display was a 22” Illyama Vision Master Pro 514 monitor set to a resolution of 1024 × 768 with a refresh rate of 60 Hz. The display was calibrated using a Minolta CS-LS110 photometer, the maximum luminance of the display was 148.33 cd/m^2^, and the minimum was 0.19 cd/m^2^. Stimuli were created and presented using MATLAB 2013b (The Mathworks, Natick) and the Psychtoolbox (Brainard, 1997; Kleiner et al., 2007; Pelli, 1997).

EEG signals were recorded using a 64-channel Active-Two Biosemi system, including eight additional facial electrodes placed on the left and right mastoids, outer canthi, supra and suborbital locations. Conductive electrode gel was used to reduce impedance. The Active-Two system uses a common mode sense and a driven right leg feedback loop to further reduce the effective impedance, please see https://www.biosemi.com/faq/cms&drl.htm for details.

### Observers

Twelve young observers reporting normal or corrected-to-normal vision took part in the EEG experiment. All participants were between the ages of 18 and 30 and biological sex was mixed. Those with photosensitive epilepsy were excluded due to the use of flickering stimuli. Ethical approval was granted by the University of Lincoln School of Social Science Ethics committee. Written informed consent was obtained from all participants prior to taking part in the study, and all experiments were conducted in accordance with the guidelines of the British Psychological Society. One observer withdrew before the end of the study, leaving data from 11 observers for analysis.

### Procedure

Observers were seated in a sound-attenuated darkened room 1 m from the display. A central white fixation cross of 1.7° visual angle appeared between each trial for 0.5 seconds. Stimuli were presented in a Gaussian-edged window with a flat area of 150 pixels and σ of 10 pixels, resulting in a viewable area of approximately 7.3° of visual angle. Observers were presented with stimuli that increased in contrast and faded to mid-gray at a rate of 5 Hz for a duration of 20 seconds each. Therefore, the average luminance remained constant throughout the image presentation. There were three repetitions of each stimulus displayed. All trials were presented in a random order anew for each individual observer. After the 20-second trial, observers were asked to rate the stimuli for discomfort on a 1–7 Likert scale. The instructions that appeared on the screen were to rate the image, “How uncomfortable is this? 1 = not 7 = very uncomfortable.” There were no additional instructions given to participants on how to interpret discomfort. Although it is accepted that “discomfort” is a multifaceted term including several factors, these are highly correlated (e.g., Sheedy et al., 2003), therefore, we chose the aggregate measure for this study in line with previous work (e.g., Marcus and Soso, 1989; Fernandez & Wilkins, 2008; Juricevic et al., 2010). It is possible that the ratings influenced the subsequent trial. However, this is mitigated by the fixation cross forcing observers to pause between making the rating and viewing the next stimulus. In addition, the trials were presented in a new random order for each participant, and so any order effects should be minimized through the averaging process.

### Analysis

EEG data were analyzed using the EEGLAB toolboxes (Delorme & Makeig, 2004). Data were rereferenced to the linked mastoids and resampled to 256 Hz offline. Data were band-pass filtered between 0.1 and 40 Hz to remove drift and line noise artifacts. Bad channels were rejected using an automatic thresholding procedure removing any channels exceeding a threshold greater than 5% probability. Missing channels were interpolated using spherical interpolation. Each 20-second epoch was extracted and a baseline of 1000 ms prior to stimulus onset was removed via subtraction. This −1000 to 0 ms baseline period was subtracted from 0 to 20,000 ms presentation duration, where 0 is the onset of the flickering stimulus. Each 20-second epoch was then further subdivided into 2-second epochs to allow for sufficient data length to perform spectral analysis but allow epochs containing substantial artifacts to be rejected. The first 2-second epoch was discarded to reduce the influence of transient effects. Epochs containing artifacts exceeding a threshold of ±500 μV were rejected. A Gratton-Coles (1983) procedure was used to correct for eye movement artifacts without needing to exclude trials. A threshold of ±20 μV in a 200 ms time window was used to define blink artifacts.

Spectral analysis was conducted using Welch’s method using the MATLAB function “pwelch,” assuming a 2-second epoch length, a sampling rate of 256 Hz, and 0 overlap. Welch’s method of spectral analysis uses a sliding window to estimate the power spectral density function. The peak of the power spectral density function at 5 Hz, the fundamental frequency of the visual stimulation, was chosen as the SSVEP response. As the stimulus faded in and out of mid-gray following a sinusoidal temporal profile, this can be considered “pattern onset/offset” SSVEP, for a technical introduction, see Norcia et al. (2015).

## Results

### Discomfort ratings

**Figure 2.**
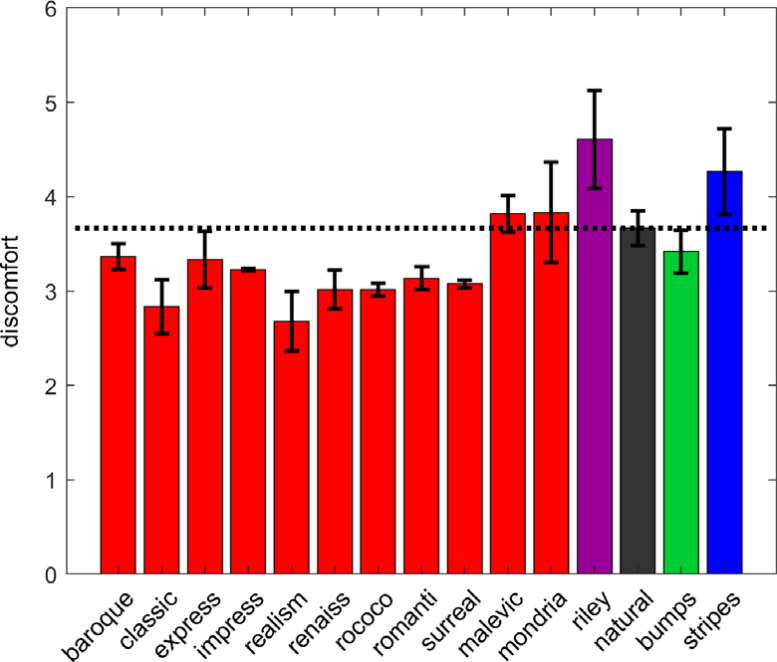
Average discomfort judgments for each of the image categories. Error bars indicate 95% confidence intervals. The black dotted line indicates the average values for the natural images to facilitate comparison across categories.

Average discomfort ratings can be seen in Fig. 2. The greatest discomfort responses are to the striped stimuli and the work of Bridget Riley. Realism results in the lowest overall discomfort response. Therefore, the discomfort judgments for the artworks are lower even though contrast measures are higher (see Supplementary Material), indicating contrast is not the sole predictor of discomfort.

We would expect some image types to be more uncomfortable than others. A linear mixed effect model was created to predict discomfort judgments including image type as a fixed effect, and observer as a random effect. There was a significant effect of image type (*F*(4,523) = 11.10, *p* < 0.001). The results can be seen in Table 2. Please note, the intercept represents the image category natural images, and this is the category to which others are compared. Compared to natural images, there was a non-significant trend for artworks to be less uncomfortable. Stripes, bumps, and the work of Bridget Riley were all more uncomfortable compared to natural images. The model accounted for 9% (adjusted *R* ^2^ = 0.09) of the variance. Please note, due to the ordinal nature of the discomfort judgments, when the assumptions were checked, this was found to show a violation of the assumptions of the linear mixed effect model. As a result, ordinal regression was used to reanalyze the data more conservatively. This showed the broadly similar pattern of results, but this time the artworks were also statistically significantly different from the natural images. Full details of the linear mixed effect model and the more conservative ordinal regression can be found in the Supplementary Material.

**Table 2.** Results of the linear mixed effect model assessing the effect of image type on discomfort judgments

Name	Estimate	SE	*T*	DF	*p*	Lower CI	Upper CI
Intercept	3.46	0.11	30.96	523	<0.001	3.24	3.68
Artworks	−0.28	0.14	−1.92	523	0.055	−0.56	0.0061
Bump	0.77	0.22	3.45	523	0.00060	0.33	1.20
Stripe	0.80	0.29	2.74	523	0.0063	0.23	1.37
Riley	0.84	0.32	2.61	523	0.0093	0.21	1.47

### EEG results

**Figure 3.**
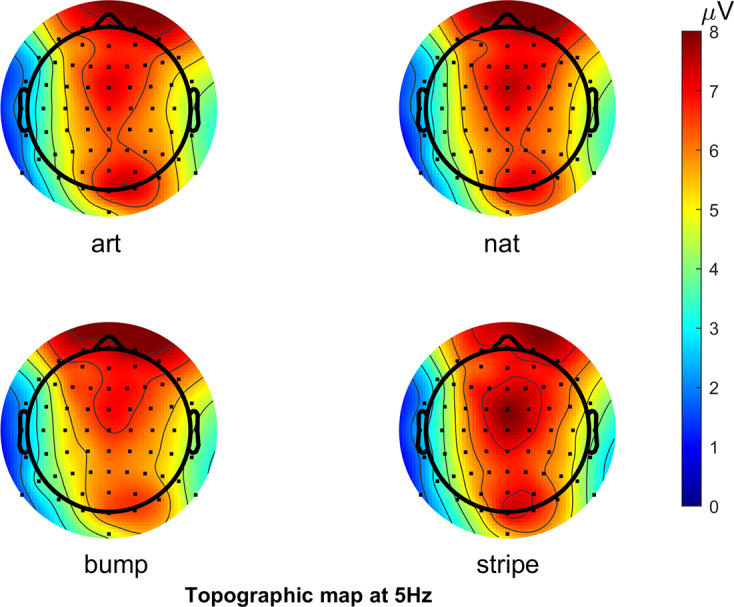
Topographic maps showing SSVEP response to fundamental frequency of 5 Hz. Note the eye channels are not included on this figure.

Fig. 3 shows the scalp topography of the SSVEP responses to each of the four image classes at the fundamental frequency of 5 Hz. Strong activity can be seen in the occipital and in the frontal channels. The occipital areas were of interest in the study based on the predictions. However, for completeness, the frontal activity was also analyzed in the same way. This can be seen in the Supplementary Material. The eye channels that do not appear in the figure were analyzed separately (see below and Supplementary Material).

**Figure 4.**
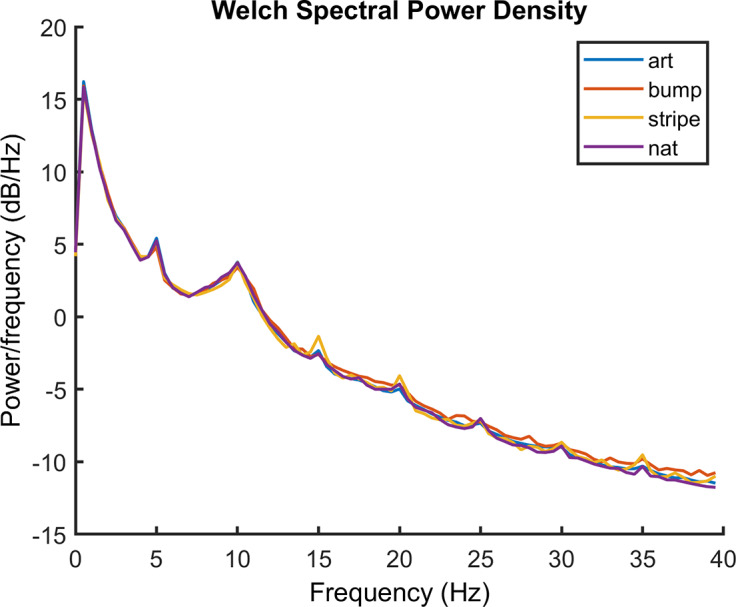
Power spectrum showing the average spectra for the response to each of the image categories: artworks, natural images, bump stimuli, and sine wave gratings, averaged over the channels of interest.

Fig. 4 shows spectral slope averaged over the channels of interest. Channels of interest were in the posterior regions and defined as Iz, Oz, O1, O2, POz, PO3, PO4, PO7, and PO8 based on the scalp topography. Clear peaks can be seen at the fundamental frequency (5 Hz) as well as the harmonics of the response. The main analysis was conducted on the fundamental frequency. For completeness, analysis of the 10 Hz harmonic was also conducted. This can be seen in the Supplementary Material. There were no statistically significant effects at the 10 Hz harmonic.

**Figure 5.**
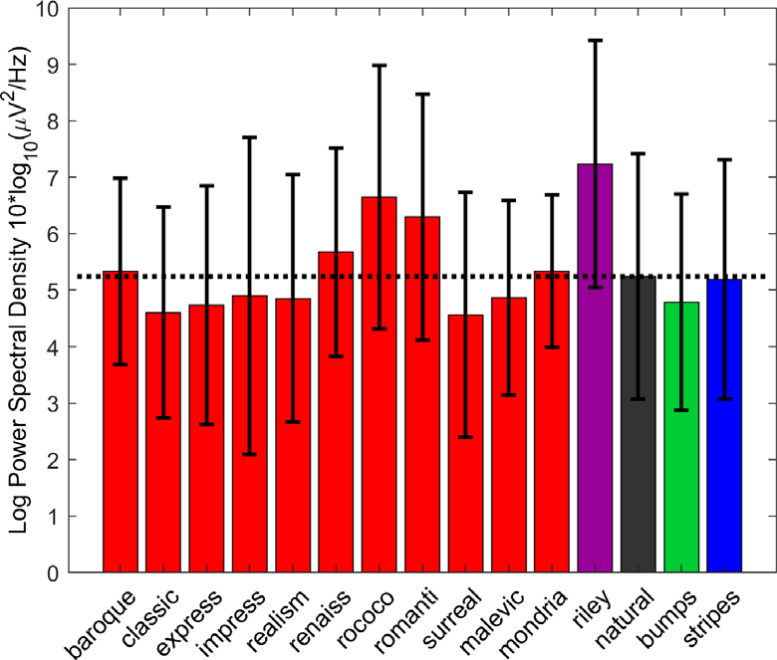
Average SSVEP response for each of the image categories. Error bars indicate 95% confidence intervals. The black dotted line indicates the average values for the natural images to facilitate comparison across categories.

Fig. 5 shows the average peak SSVEP response at the fundamental of 5 Hz for each of the stimulus categories. Typical of SSVEP responses, peak responses can be seen at the fundamental frequency of stimulation (5 Hz), and the harmonics.

Fig. 5 shows the average SSVEP response for all images within a category.

Analysis of these SSVEP results, and their relationship to discomfort ratings, model responses, and image statistics, are presented below in relation to the hypotheses outlined in the introduction:

Hypothesis 1.
Can we predict discomfort judgments from SSVEP and total model responses?

**Figure 6.**
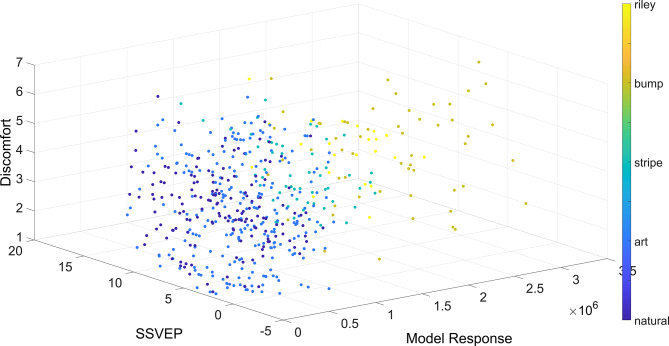
Discomfort judgments predicted by SSVEP and total model responses, each color indicates a different image category.

A linear mixed effect model was created to predict discomfort judgments from SSVEP, total model response and model response kurtosis, taking image category and observer as random variables. The model accounted for 8% of the variance in discomfort judgments (*R* ^2^ = 0.08). Discomfort judgments were predicted by total model response (estimate of the coefficient = 4.84x10^−7^, SE = 1.43x10^−7^, *p* < 0.01, CI = [2.03 x10^−7^, 7.64 x10^−7^]), and model response kurtosis (coefficient = 0.003, ± 0.001 SE, *p* < 0.05, CI = [0.0005, 0.005]), but not SSVEP responses (coefficient = −0.004, SE = 0.02, *p* = 0.83, CI = [−0.04, 0.03)). Fig. 6 shows a scatterplot of discomfort judgments predicted by SSVEP and total model responses, image category is indicated with the different colors. For assumptions of the model, the model fitting process, and the complete set of outputs of the model, please see Supplementary Material.

Hypothesis 2.
Are the smallest SSVEP responses elicited by artworks, and the largest responses by grating and bandpass filtered noise?

Linear mixed effect model was created to predict SSVEP responses from image type, including observer as a random intercept and random slope. There was a significant effect of image type (*F*(4,523) = 3.37, *p* = 0.01). Compared to natural images, only the work of Bridget Riley elicited a statistically significant greater SSVEP response. Again, please see Supplementary Material for more detail on the model fitting process and full details of outputs.

Hypothesis 3.
Do the mid-range spatial frequencies elicit the greatest discomfort and largest SSVEP responses?

**Figure 7.**
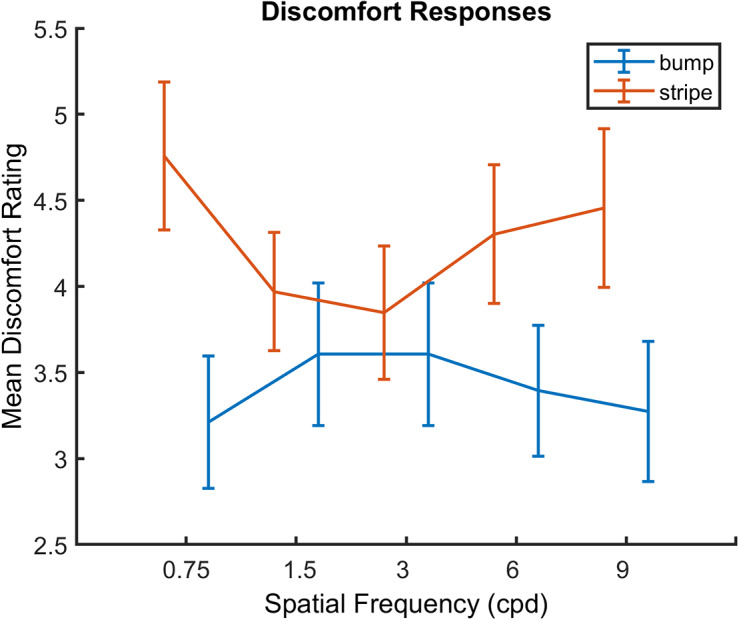
Spatial frequency tuning of discomfort responses, error bars are ±1SE of the mean.

Based on previous literature, we would expect discomfort judgments to show spatial frequency tuning in those images that vary systematically by spatial frequency (bumps and stripes). Fig. 7 shows spatial frequency tuning for discomfort judgments. This appears to be in a different direction for bump and stripe stimuli, which is unexpected, based on previous work. A linear mixed effect model including spatial frequency (as both quadratic and linear terms), image type (bump or stripe), and their interaction was created, including the observer as a random effect (slope and intercept). As the functions contain a maximum or minimum, rather than a monotonic relationship, spatial frequency was included as a quadratic term. Further, as this peak is by necessity at a non-zero spatial frequency, a linear term for spatial frequency was also included to allow us to capture tuning around this center frequency. Results showed there to be a significant linear effect of spatial frequency (indicating non-zero peak, coefficient = −0.82, ± 0.39 SE, *p* = 0.040, CI = [−1.62–0.04]), a significant quadratic effect of spatial frequency (indicating tuning, coefficient = 0.15, ± 0.07 SE, *p* = 0.025, CI = [0.02 0.28]), and a significant interaction between spatial frequency and image type (coefficient = −0.06, ± 0.02SE, *p* = 0.003, CI = [−0.11–0.02]). To unpack this interaction, two separate mixed effects models, including spatial frequency as both a linear and a quadratic term showed there to be no significant effects of spatial frequency for either bump or stripe stimuli. No significant effects of spatial frequency were found for this quadratic, reflecting the only modest spatial frequency tuning evident for each stimulus type in Fig. 7. For full details of the model, please see Supplementary Material.

**Figure 8.**
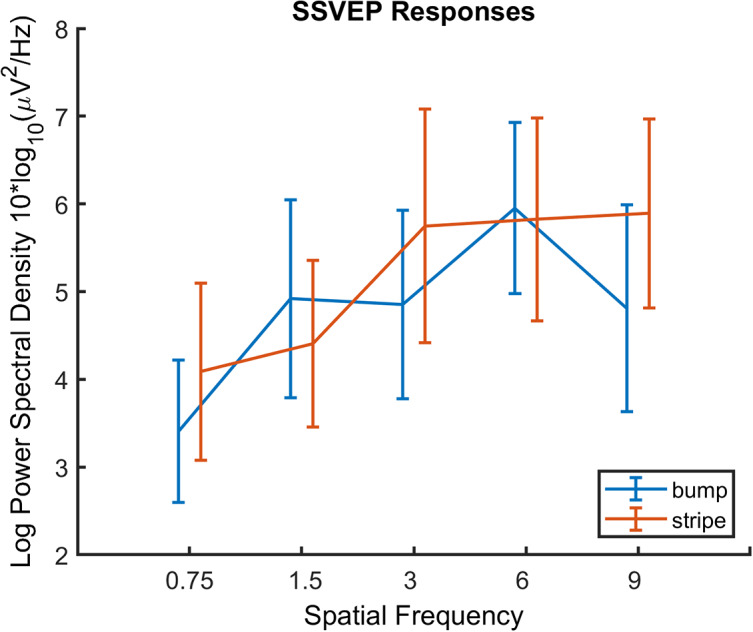
Spatial frequency tuning of SSVEP responses, error bars are ±1SE of the mean.

We would expect the SSVEP response to vary with spatial frequency for images where this has been systematically varied, specifically bumps and stripes. This effect of spatial frequency is clear in Fig. 8, which shows SSVEP increasing with frequency. While we might predict a peak at midrange frequencies when image size is kept constant, it should be noted that the spatial contrast sensitivity tends toward a more lowpass character at higher temporal frequencies, as used here (Kelly, 1979). Due to the monotonic effect of spatial frequency on SSVEP, a linear mixed effect model was created for the bump and stripe images, including spatial frequency and image type as fixed effects, with observer as a random effect (slope and intercept). This allowed us to model the increase in SSVEP with increasing spatial frequency. Our prediction was supported, Fig. 8 shows the increase in SSVEP power with increasing spatial frequency (coefficient = 0.44, ± 0.20 SE, *p* = 0.026, CI = [0.06 0.83]). This is in itself as expected, as the SSVEP response has long been demonstrated to show spatial frequency tuning (e.g., Plant, 1983). However, it does demonstrate that the manipulation is working as expected. For full details of the model, please see Supplementary Material.

Hypothesis 4.
Do low-level image statistics predict discomfort judgments and neural responses?

**Figure 9.**
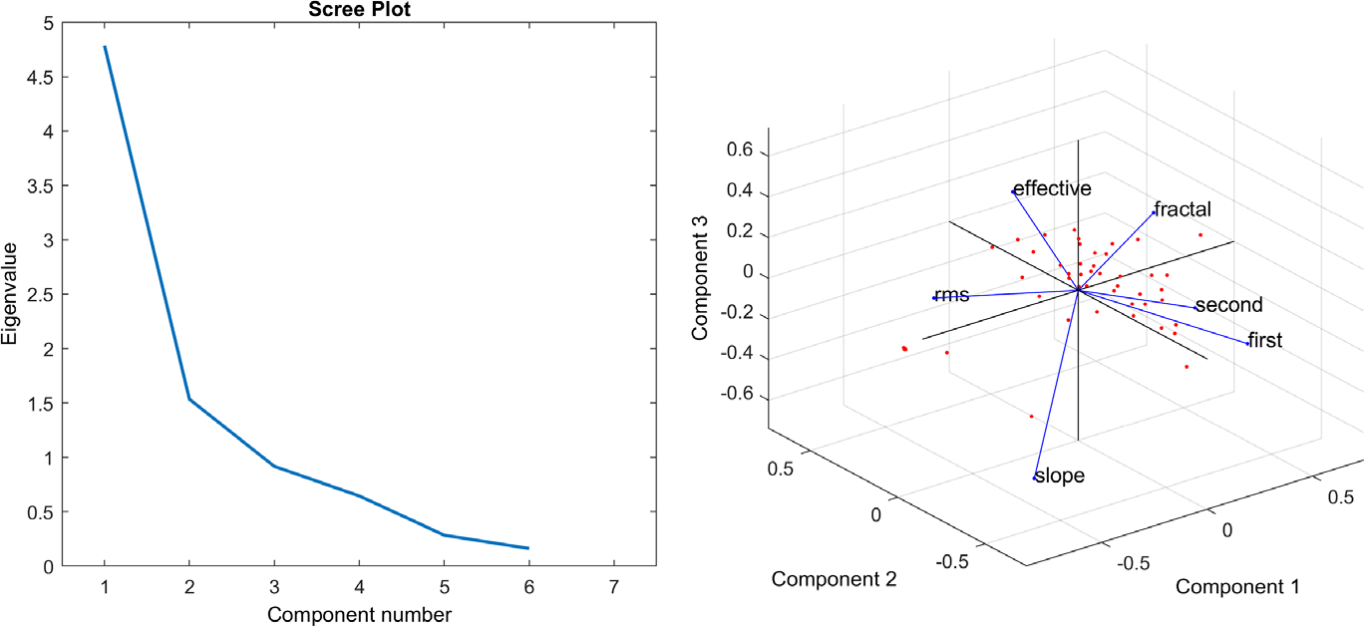
Left: Scree plot of the eigenvalues against component number and Right: PCA loadings. “First” refers to first-order edge orientation entropy, “second” refers to second-order edge orientation entropy, “fractal” refers to fractal dimension, “effective” refers to effective contrast, “RMS” refers to root-mean-squared contrast, and “slope” refers to spectral slope.

Image statistics are highly non-independent. For example, RMS and CSF-filtered contrast are both related measures of contrast in the image, first-order edge orientation entropy is closely related to second-order edge orientation entropy. To reduce dimensionality, principal component analysis was conducted including image statistics of fractal dimension, spectral slope, RMS and CFS-filtered contrast, the total model response, first- and second- order edge orientation anisotropy. The first three principal components accounted for much of the variance, the first component accounted for around 57% of the variance, and the second component accounted for around 18% of the variance, and the third around 11% of the variance. Only the eigenvalues for principal components 1 and 2 were greater than 1, however principal component 3 was also included in the analysis following testing the assumptions and the model build statistics (see Supplementary Material). Fig. 9 shows the scree plot and the loadings.

A linear mixed effect model was created to predict discomfort judgments from the principal components 1, 2, and 3, including the observer as a random effect. Principal component 1 significantly predicted discomfort judgments (−0.12, ± 0.03 SE, *p* < 0.001, CI = [−0.17, −0.06]), as did principal component 3 (−0.24 ± 0.07 SE, *p* < 0.05, CI = [−0.37–0.11]). Principal component 2 did not predict discomfort judgments statistically significantly (−0.08, ± 0.05 SE, *p* = 0.10, CI = [−0.18, 0.02]). However, the model accounted for a negligible amount of the variance (6%). This did not meet the requirements of the linear mixed model, and so again ordinal regression was used as a more conservative estimate, this gave a similar pattern of results, please see Supplementary Material for model build, assumptions, and alternative analysis.

A linear mixed effect model was created to predict the SSVEP response from principal components 1 and 2, including observer as a random effect. The model accounted for 58% of the variance (adjusted *R* ^2^ = 0.58). Principale component 1 was not statistically significant (coefficient = 0.05, ± 0.05 SE, *p* = 0.34, CI = [−0.05 0.14]), but principal component 2 was statistically significant (coefficient = 0.18, ± 0.09 SE, *p* < 0.05, CI = [0.01 0.35]), as was principal component 3 (coefficient = −0.61, ± 0.10 SE, *p* < 0.05, CI = [−0.82–0.39].

The first principal component loadings were low slope, high first order entropy and high second order edge entropy values. Low slope values will relate to images with relatively less low-spatial frequency information compared to high spatial frequency information, perhaps images that include more fine details. The first and second order edge orientation entropy values relate to the predictability of the edge information in images. Therefore, the first principal component might be interpreted as relating to edge predictability in highly detailed images, and unstructured, highly detailed images are uncomfortable. The second principal component loadings were low fractal dimension, high RMS and high CFS-filtered contrast values. Images low in fractal dimension have less predictability in the form of self-similar patterns compared to images with high fractal dimension, thus the second principal component might be interpreted as relating to images with high contrast, but low predictability. The third principal component related to high fractal dimension, low slope, and low first order edge orientation entropy. This might be interpreted as complex, predictable images with predominantly more fine edge information. This showed a negative relationship with both discomfort judgments and SSVEP responses.

The stripes were removed from the principal component analysis due to undefined values for slope. However, the relationship between RMS contrast and discomfort can be assessed in the whole image set. Considered separately, RMS contrast does predict discomfort judgments (coefficient = 3.32 ± 1.3 SE, *p* = 0.01, CI = [0.75 5.89]), but again only a small amount of the variance is accounted for (9%). Similarly, when striped patterns are included, CFS-filtered contrast can predict discomfort judgments (coefficient = 0.02 ± 0.01SE, *p* = 0.03, CI = [0.002 0.04]), with 9% of the variance explained. Please see Supplementary Material for full details of the models.

### Control analysis: Eye channels

It has been suggested that visual discomfort from flicker may be related to eye movements (Kennedy & Murray, 1991; Wilkins, 2016). Although the flicker in the literature tends to be much faster than the rates used in the current study, given that there was a large response in the frontal electrodes, particularly for the 5 Hz SSVEP responses, it was considered important to check for this possibility. Therefore, vertical and horizontal eye channels were estimated following Jia and Tyler (2019), full analysis can be seen in the Supplementary Material. There was no statistically significant relationship between the horizontal (*p* = 0.88) or vertical (*p* = 0.38) electrodes with discomfort judgments of stimuli. Neither the horizontal (*p* = 0.51) nor vertical electrodes (*p* = 0.19) showed a significant relationship with spatial frequency for the stripe and bump stimuli. Neither PCA1 nor PCA2 predicted the horizontal eye channels (*p* = 0.86, *p* = 0.41, respectively) nor the vertical eye channels (*p* = 0.15 and 0.99, respectively).

## Discussion

The aim of the current study was to investigate the predictions of efficient coding in response to artworks, natural images, and uncomfortable images. Our specific goals were to understand (1) which factors predict discomfort, (2) the relationship between discomfort and SSVEP responses, (3) how SSVEPS vary across image categories, (4) the effects of spatial frequency on SSVEP and discomfort, and (5) the relationship between image statistics, SSVEPs and visual discomfort, via dimension reduction. We used a model of early visual processing, SSVEP responses, and image statistical properties such as contrast, fractal dimension, spectral slope, and edge information entropy. As these statistical properties are highly interrelated, principal components analysis was performed to reduce the dimensionality into fewer components. Two major components emerged the first might be interpreted as relating to the presence of unstructured, high spatial frequency information, and the second to contrast. A third component also emerged, related to higher fractal dimension, low spectral slope and low first-order edge entropy; this component showed a negative relationship with both discomfort and SSVEP responses.

As in previous work, uncomfortable images had statistical properties that differed from natural images (Fernandez & Wilkins, 2008; Juricevic et al., 2010; O’Hare & Hibbard, 2011). Striped patterns and the work of Bridget Riley were judged the most uncomfortable, in agreement with previous research (O’Hare, 2017a; O’Hare et al., 2021; Wilkins et al., 1984). Artworks showed a non-significant trend toward being more comfortable compared to natural images, again in line with the predictions that artworks might be pleasing to the eye (e.g., Graham & Field, 2007b; Graham & Field, 2008).

Importantly, discomfort judgments were predicted by the computational model of early visual processing. This supports previous modeling work (Hibbard & O’Hare, 2015) suggesting that low-level, feed-forward visual processing can account for some aspects of visual discomfort in a wide range of images, including different genres of artworks and different types of artificial images thought to be uncomfortable. Moreover, spectral slope and first and second order edge anisotropy are both related to principal component 1, which predicted discomfort, suggesting that unstructured, highly detailed images were those judged more uncomfortable.

Unstructured highly detailed images might prove challenging for the visual system as they contain a lot of unpredictable visual information. Predictable visual information could be efficiently processed, and this is determined by their structure (Field, 1999). Although the focus of this study is visual discomfort, the converse argument is that images with a predictable structure should be esthetically pleasing. Edge properties are important for esthetics (Grebenkina et al., 2018; Stanischewski et al., 2020). Several of the metrics of image statistical properties are determined by edge information, for example, edge orientation anisotropy; the higher the entropy, the less predictable the orientations of the edges in the image are (Redies et al., 2017). Although spectral slope does not directly take account of edge locations, it does reflect the level of detail and self-similarity of an image (Graham and Redies, 2010). Spectral slope has been directly associated with visual discomfort (Juricevic et al., 2010; O‘Hare & Hibbard, 2013).

It is important to note that discomfort will be an aggregate of several components, including image contrast, composition, illusory effects, and semantic content. This explains why the linear mixed effects models did not account for a large amount of the variance of discomfort judgments, despite statistically significant predictors. There are several contributing concepts to visual discomfort, including blurring, eyestrain, and headache (Sheedy et al., 2003), reflected in the variability of questions used to measure discomfort, including topics related to illusory percepts and the readability of text (Conlon et al., 1999; Wilkins & Evans, 2001). In addition, high-level and semantic processes influence discomfort judgments for real images, and there is less experimental control over image content. For example, natural scenes containing buildings may be problematic as some architecture styles have been associated with discomfort (Alkhalifa et al., 2020; Le et al., 2017). However, generalizability to real images was an important consideration in the current work.

Principal component analysis did not use the stripe images, as there is no spectral slope value for these images, nor is there a value for first and second order edge anisotropy for these stimuli. When stripes are included, RMS and CFS-filtered contrast predict discomfort judgments. Neural responses to images measured using SSVEP were predicted by principal component 2, but discomfort judgments were not. This component consisted of low fractal dimension, high RMS- and CFS-filtered contrast. Fractal dimension is a measure of image predictability and complexity, and repeating self-similar patterns have been suggested to be easier to process for the visual system (Aks & Sprott, 1996; Spehar et al., 2003). Images low in fractal dimension lack this predictable structure, and so arguably may be less easy for the visual system to process. CFS-filtered contrast is determined by the modulation transfer function in conjunction with the image spatial frequency content. Contrast and spatial frequency content are important to both SSVEP responses (Plant, 1983) and discomfort judgments (Fernandez & Wilkins, 2008), however, behaviural results show that discomfort judgments are not altered when contrast effects are accounted for (O’Hare & Hibbard, 2011). In a sophisticated model including contrast normalization processes, Penacchio et al. (2023) have shown that model activation, sparseness, and isotropy all relate to visual discomfort. Overall, this result further strengthens the idea that discomfort cannot be entirely accounted for by simple contrast effects, although the neural responses are heavily influenced by global image contrast for a wide range of artworks, natural images, and artificial images.

Discomfort judgments and SSVEP responses were negatively related to principal component 3, that related to high fractal dimension, low spectral slope, and low first-order edge entropy (highly detailed, predictable images). Many naturally occurring fractal patterns are highly detailed (Spehar et al., 2003). Recent work has shown fractal patterns to influence walking speed (Burtan et al., 2023) supporting the idea that the visual system is optimized to process the kinds of images encountered in nature.

Discomfort judgments showed spatial frequency tuning. For striped stimuli, this is in the expected direction, with mid-range spatial frequencies being the more uncomfortable. By contrast, mid-range bump stimuli were judged to be the most comfortable, which is not in line with previous results (Fernandez & Wilkins, 2008; O’Hare & Hibbard, 2011). This is unexpected, but is perhaps due to stimulus flicker, since in the previous experiments mid-range bump stimuli were shown to be more uncomfortable in static images. Bump stimuli have been shown to be more uncomfortable than other image categories in previous work using SSVEP responses (O’Hare et al., 2021), although this study did not address spatial frequency tuning.

It was unexpected that SSVEP responses did not predict discomfort judgments. It could be argued that the eye movements may account for visual discomfort in the current study, especially given the strong response in the eye channels. Additionally, observers may include effects relating to eye movements in their assessment of discomfort. For example, the Pattern Glare test specifically refers to shimmering and scintillating illusions in the static image (Wilkins & Evans, 2001), however, these patterns remain uncomfortable even in the absence of eye movements (O’Hare, 2017b). Works of Op-art designed to induce illusory motion effects have been investigated in terms of illusory motion (e.g., Otero-Millan et al., 2012; Troncoso et al., 2008) although this was not found to relate to eye movements (Hermens & Zanker, 2012). The Supplementary Material shows the eye movement analysis for the current study. There is no distinguishable SSVEP response in the eye channels, and no systematic relationship is found with the eye channel responses. Therefore, although effects relating to eye movements may have contributed to the appraisal of discomfort, eye movements alone cannot account for the results in the current study. In future research, it would be helpful to measure eye movements directly. It has been shown in the past that relationships between SSVEP responses and discomfort judgments are relatively small (O’Hare et al., 2021). As the SSVEP response is heavily influenced by image contrast (e.g., Plant, 1983) and physical contrast was allowed to vary in the current study, a parsimonious explanation is that any relationship between SSVEP and discomfort might be overwhelmed by effects of physical contrast.

We used a diverse range of images, which by necessity creates a lack of experimental control of many image properties. Color was not included to allow for greater comparability between images, but plays an important role in visual discomfort (e.g., Penacchio et al., 2021). It may also be beneficial in future to investigate the effects of edges more systematically using parametrically controlled stimuli. Importantly, from the current study, it seems that the predictability of images is important in visual discomfort, as predicted by efficient coding. Edge information is carried in the phase spectrum, which can be scrambled (e.g., Coggan et al., 2016) or swapped between images (e.g., Oppenheim & Lim, 1981). In summary, visual discomfort for a wide range of image types, including varying art genres, natural and artificial images, was predicted by a low-level model of visual discomfort. Low-level image statistics relating to highly detailed, unstructured images predicted discomfort judgments, whilst neural responses measured using SSVEP were predicted predominantly by contrast. This provides support for the ideas of efficient coding in accounting for some aspects of visual discomfort.

## Supporting information

O’Hare and Hibbard supplementary material O’Hare and Hibbard supplementary material

## Supplementary material

The supplementary material for this article can be found at http://doi.org/10.1017/S0952523824000051.
