# Supplementary material for: Support for the efficient coding account of visual discomfort
Source: Vis Neurosci. 2024 Dec 26;41:E008. doi: 10.1017/S0952523824000051 (PMC11730975; doi:10.1017/S0952523824000051)
Supplement: O’Hare and Hibbard supplementary material [file S0952523824000051sup001.docx]

**Supplementary Information**

**1) Additional Figures**

Computational Model

Total model response can be seen in Supplementary Figure 1. The greatest total response was for striped images and the work of Bridget Riley. Bump stimuli also result in high overall response. The lowest responses were for abstract artwork, the work of Malevich and Mondrian, and for the other groups of art. Natural images had some of the lowest total response. This indicates that the computational model is swayed by contrast of the image, however the composition of the image also plays a part, as this is not a direct relationship between the contrast and the model response.


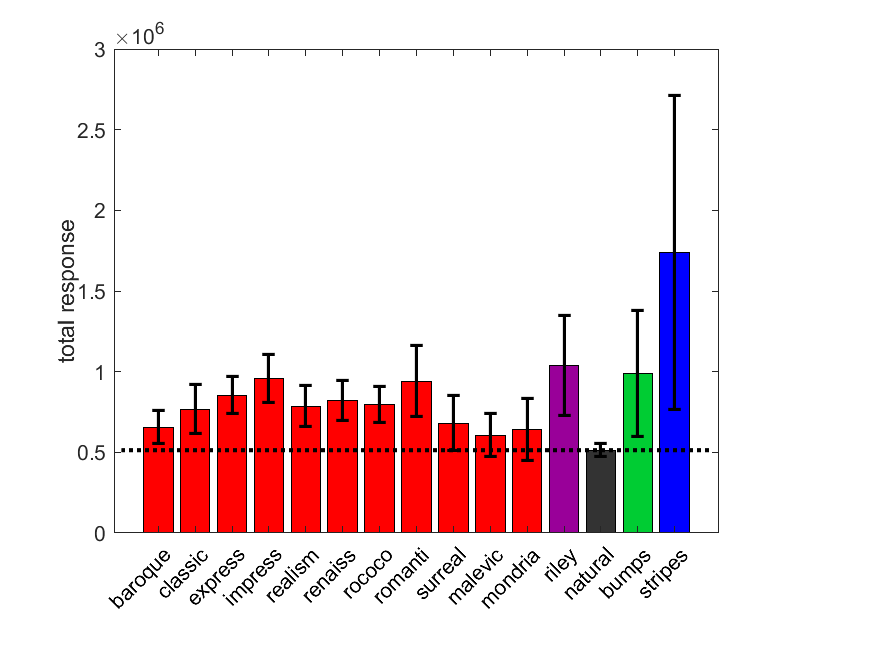


*Supplementary Figure 1: Total model response averaged over each of the image categories. Error bars indicate confidence intervals. The black dotted line indicates the average values for the natural images to facilitate comparison across categories.*

Supplementary Figure 2 shows the model response kurtosis. There was high kurtosis for some of the artwork genres, including work of Surrealism, and that of Malevich and Mondrian. There was low kurtosis overall for the bump stimuli and many of the artwork genres.


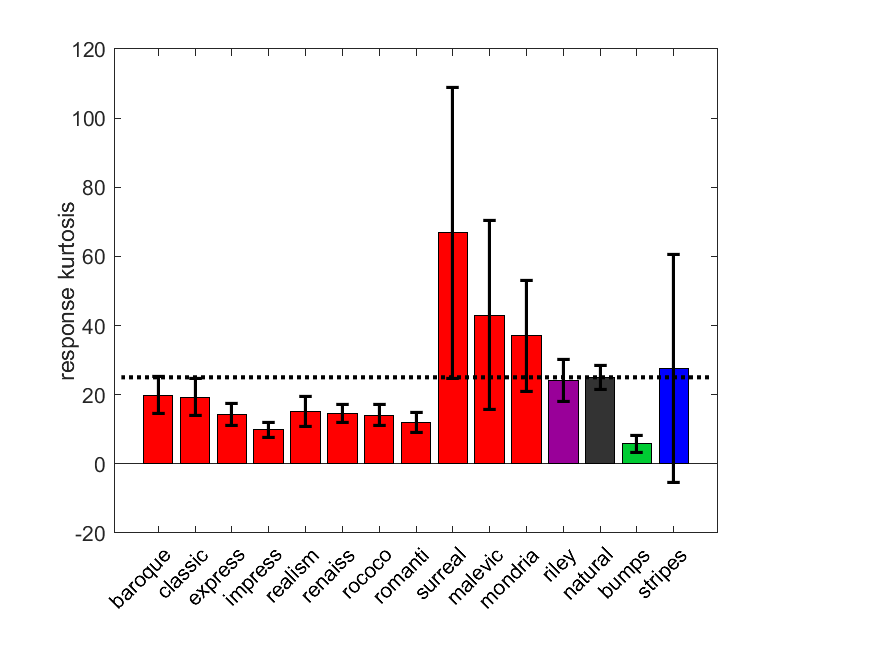


*Supplementary Figure 2: Model response kurtosis averaged over each of the image categories. Error bars indicate confidence intervals. The black dotted line indicates the average values for the natural images to facilitate comparison across categories.*

Image statistics

Fractal dimension

Fractal dimension can be seen in Supplementary Figure 3. In general, the fractal dimension for artworks and uncomfortable stimuli is very similar to that for natural images. The exceptions are that the fractal dimension values are lower for the work of Bridget Riley and Impressionist artworks. This lower fractal dimension for these two classes of artworks indicates lower image complexity than for photographs of natural scenes.


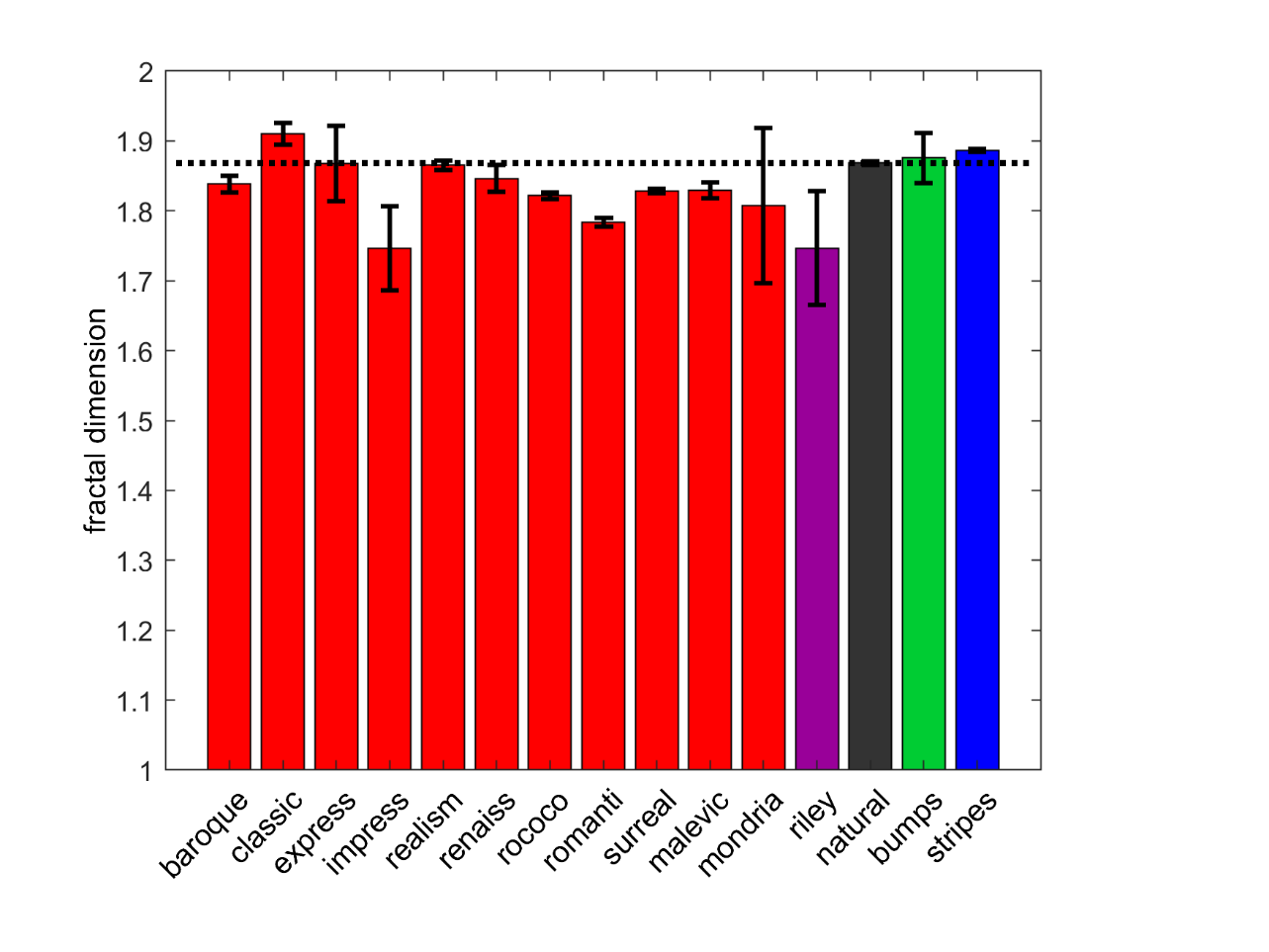


*Supplementary Figure 3: Average fractal dimension for each of the image categories. Error bars indicate confidence intervals. The black dotted line indicates the average values for the natural images to facilitate comparison across categories.*

Spectral slope

Spectral slope estimates for each image type can be seen in Supplementary Figure 4. As expected, spectral slope values for natural images average around -1. This is within the range generally reported for natural images, between -0.8 and -1.5 (Tolhurst, Tadmor and Chao ,1992), although the natural images in this set are within the lower end of this range. The spectral slope values for the works of Bridget Riley are much closer to 0, indicating more equal contrast across scale than is found in natural images in this sample. Most other artworks are within the range typical of natural images identified by Tolhust et al., (1992), with the exception of the work of Mondrian, which is steeper than expected. This indicates a rapid drop-off in amplitude with scale, such that the relative contrast at high spatial frequencies is lower than is found for natural images.


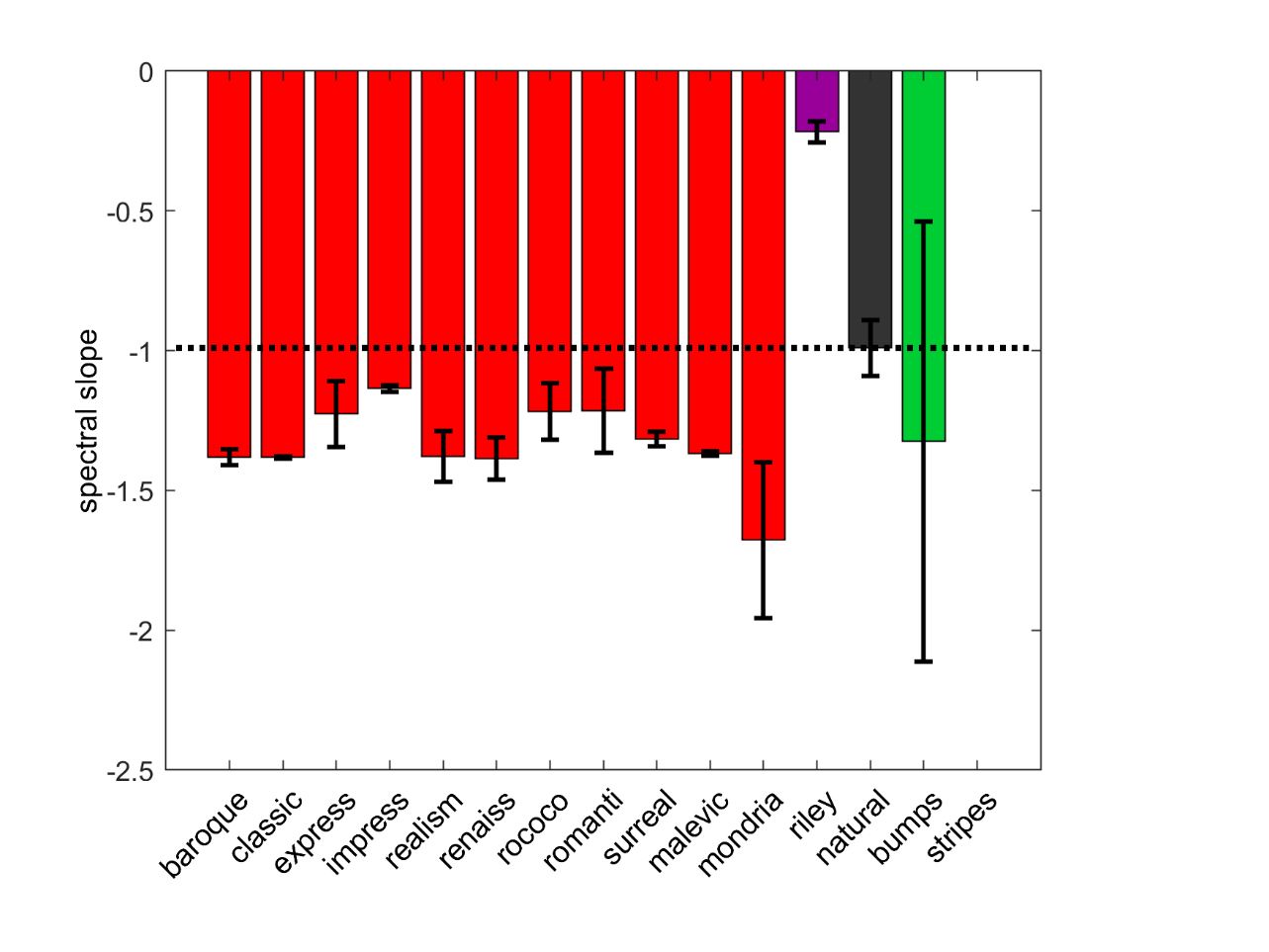


*Supplementary Figure 4: Average spectral slope for the image categories. Error bars indicate confidence intervals. The black dotted line indicates the average values for the natural images to facilitate comparison across categories.*

RMS contrast

Supplementary Figure 5 shows RMS contrast across image categories, which is notably higher for the work of Bridget Riley as well as the striped images. This contrast is higher than the photographs, as this was the linear image set used, and so might have been lower in overall contrast compared to the log-transformed natural image set.


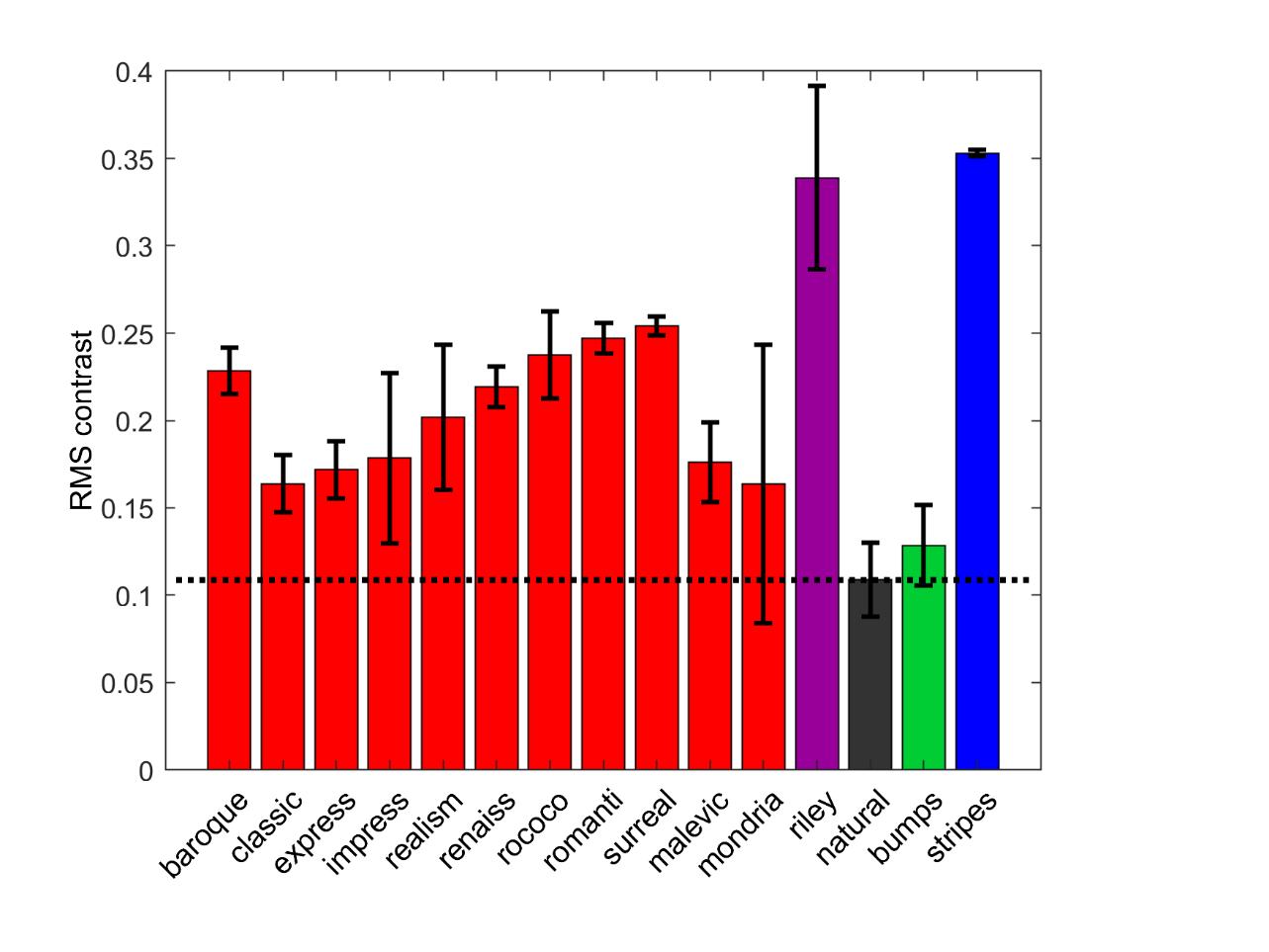


*Supplementary Figure 5: Average RMS for each of the image categories. Error bars indicate confidence intervals. The black dotted line indicates the average values for the natural images to facilitate comparison across categories.*

Estimated perceived contrast

The maximum estimated perceived contrast (using the modulation transfer function) was for the romantic artworks (Supplementary Figure 6). Stripes have the lowest estimated perceived contrast. An independent t-test showed artworks to have a greater effective contrast compared to natural images (t(36) = 4.53, p < 0.001). Again, the artworks are higher contrast compared to the natural images used, possibly because of the linear version of the images was chosen, rather than the log-transformed ones.


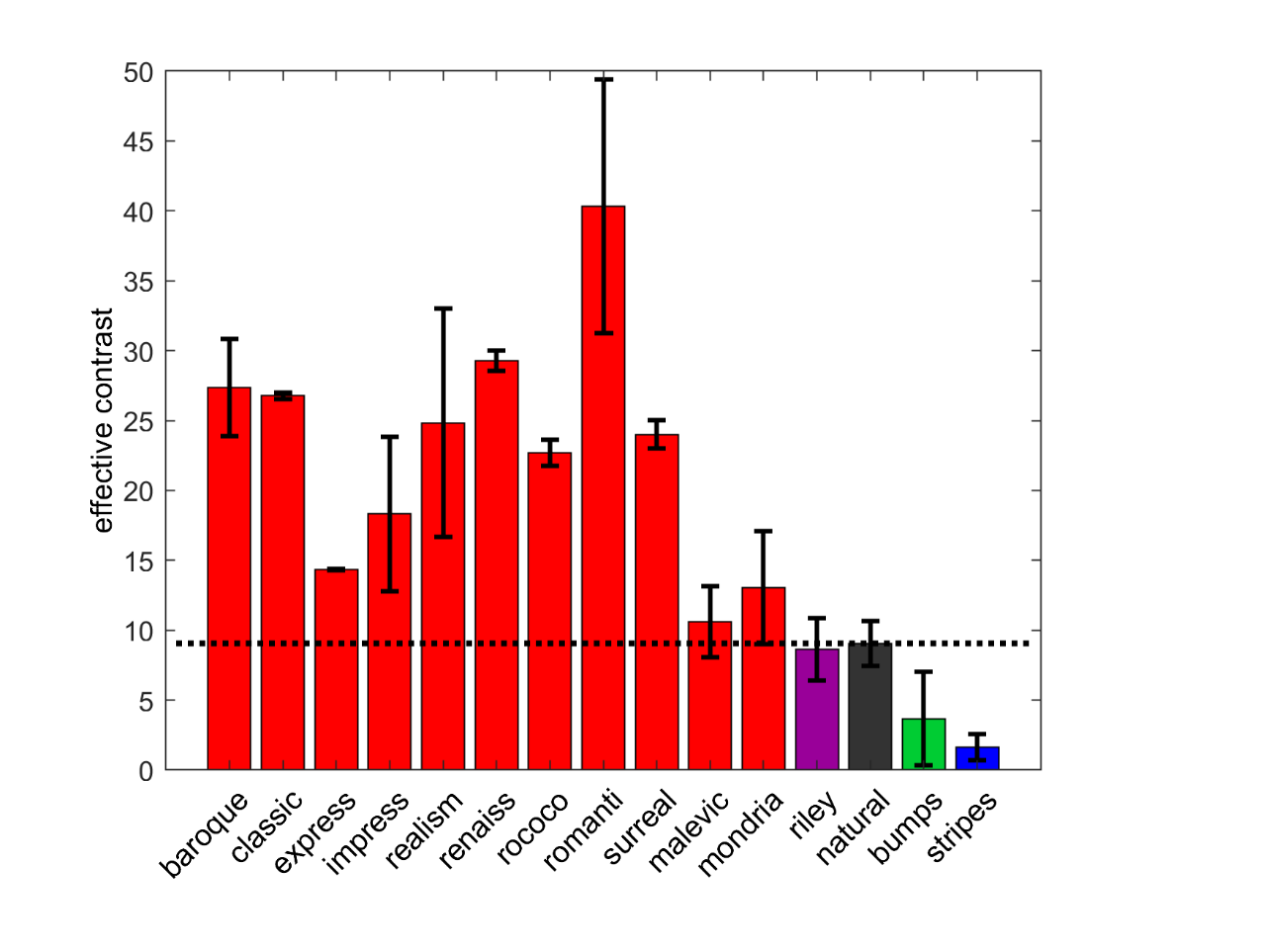


*Supplementary Figure 6: Average estimated effective contrast for each of the image categories. Error bars indicate confidence intervals. The black dotted line indicates the average values for the natural images to facilitate comparison across categories.*

Edge orientation entropy

Average Shannon entropy values for each image type can be seen in Supplementary Figure 7. As there is only one orientation for the sine wave gratings, there is no value for Shannon entropy for these images. The higher the Shannon entropy value, the more even the distribution of edge orientations in the image. Bump stimuli have the highest Shannon entropy values, reflecting their isotropic amplitude spectrum. Perhaps unsurprisingly, Mondrian images have the lowest Shannon entropy values, indicating the least even distribution of edges. This reflects the strong adherence to the use of only horizontal and vertical lines in his abstract works (Taylor, 2004). Other genres of artwork appear to be close to the entropy values of the natural images, but Surrealism, and the work of Bridget Riley, are lower. The histograms of the edge information (differences in orientation) for the two Surrealist images, the two works of Bridget Riley, the two works of Piet Mondrian, and the first two natural images (for comparison) can be seen in Supplementary Figure 8. These are not quite comparable to the edge orientations shown in Redies et al., (2017) as the current plot is of the edge differences. However, in a similar way to the work of Redies et al., (2007) it can be shown that the images consisting of many vertical and horizontal lines (e.g. the work of Riley and Mondrian) results in a considerably “spikier” distribution compared to the natural images, as shown by Redies et al., (2007).


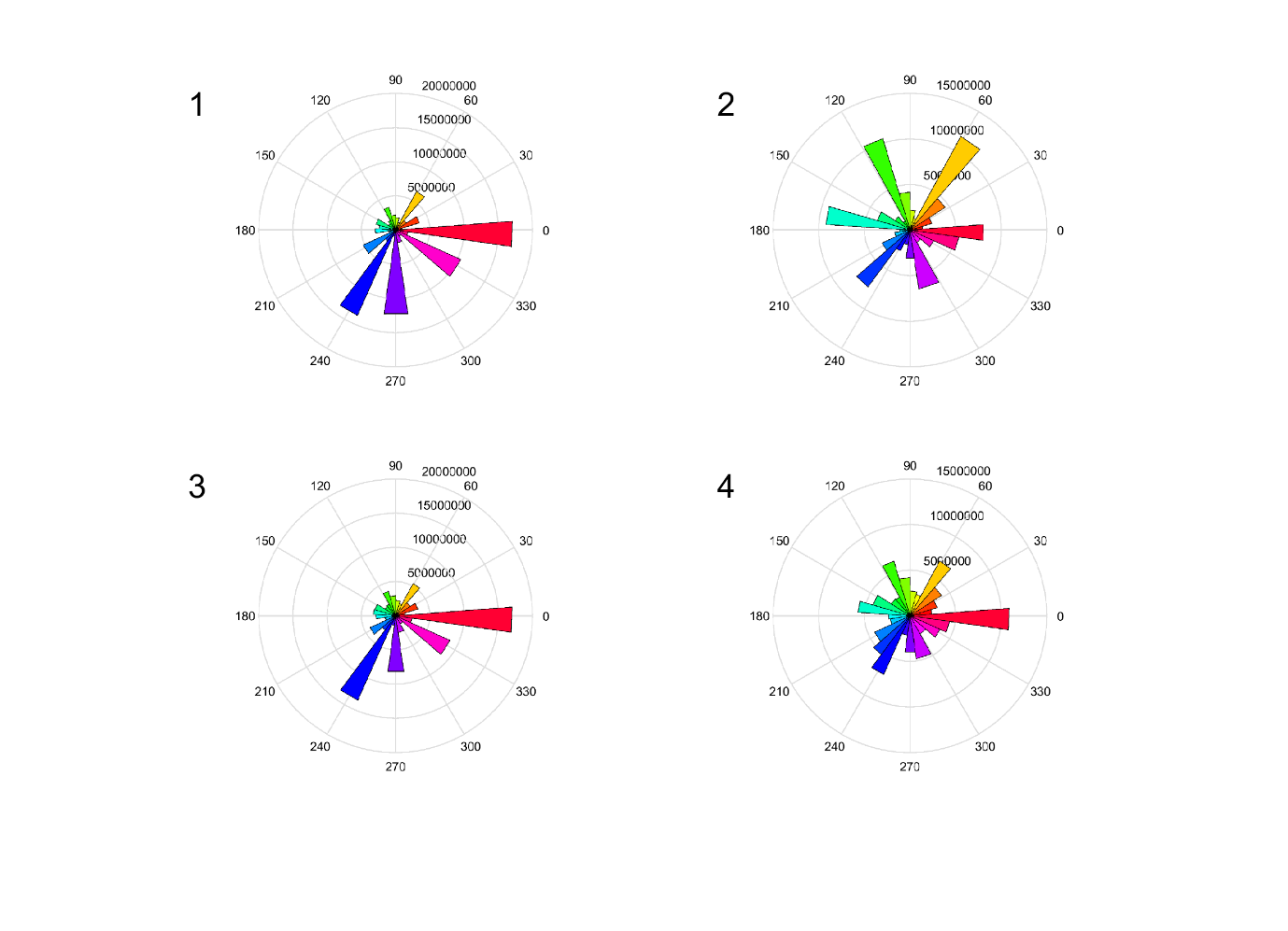


*Supplementary Figure 7: Histograms showing the distribution of orientation differences for 1) the work of Piet Mondrian, 2) the work of Bridget Riley, 3) the Surrealist works, 4) the two first natural images used in the data set.*


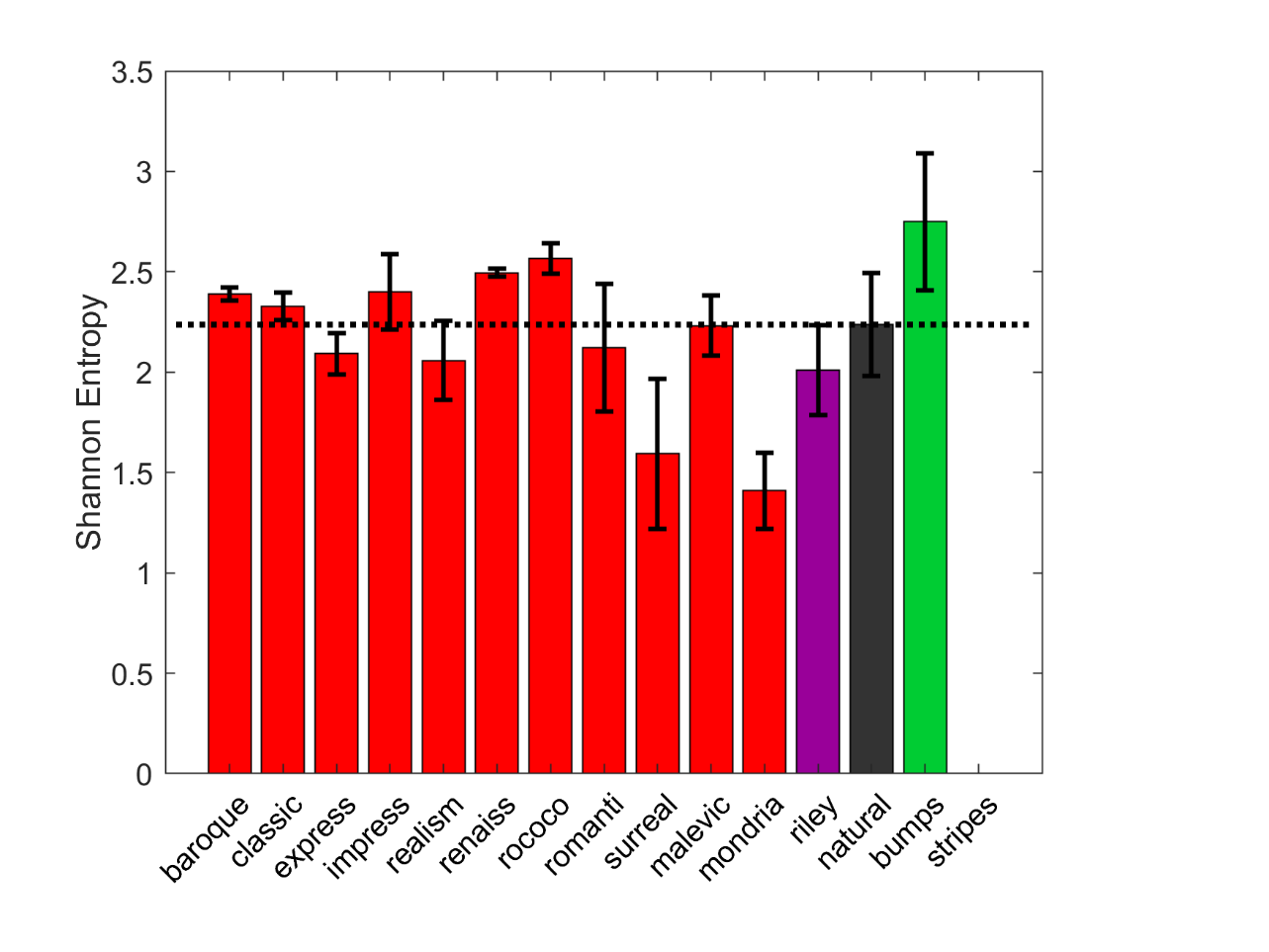


*Supplementary Figure 8: Shannon entropy values averaged over image category. Error bars indicate confidence intervals. The black dotted line indicates the average values for the natural images to facilitate comparison across categories.*

**2) Model Fits and Assumptions**

Assumptions of the linear mixed models were assessed by inspecting the relevant figures. In the following section this will be assessed one at a time for each of the models fitted.

Model equation: Discomfort ~ SSVEP + model + kurtosis + (1|imagetype) + (1+SSVEP|obs).

Supplementary Figure 9 shows the assumptions of the first model fit. The residuals are broadly speaking normally distributed, with some slightly heavier tails. This is typical of datasets and the model is robust to minor violations of normality (Schielzeth et al., 2020). The distribution of residuals against fitted values does not show any additional patterns or trends.

Supplementary Table 1 shows the building of the model and comparison with simpler models. Supplementary Table 2 shows the complete model output. As there is a reasonable concern that the variables might be correlated, pairwise correlation coefficients were estimated for the variables total model response and kurtosis (r = -0.365), which is relatively weak in this context.

Model building process: Discomfort ~ SSVEP + model + kurtosis + (1 | image type) + (1 + SSVEP | observer)

| Model specification | Model name | Nested /simpler model | Fixed effects added |  | Random Effects | | Model Fit | | | | | LRT Test | | | |
| --- | --- | --- | --- | --- | --- | --- | --- | --- | --- | --- | --- | --- | --- | --- | --- |
|  | | | |  | Subjects | Items | AIC | BIC | LL | df | df | | Χ^2^ | p |  |
| RE only | Null | - | - |  | intercepts | intercepts | 1882.9 | 1908.5 | -935.4 | 6 |  | |  |  |  |
| FE main effects | Main effects 1 | Null | SSVEP + model + kurtosis |  | intercepts | Intercepts | 1874.9 | 1904.8 | -930.4 | 7 | 1 | | 9.99 | 0.001 |  |
| FE main effects | Main effects 2 | Null | SSVEP + model + kurtosis |  | intercepts | None | 1880.4 | 1906 | -934.2 | 6 | 0 | | 2.47 | 0.000 |  |
| **FE main effects** | **Main effects 3** | **Null** | **SSVEP + model + kurtosis** |  | **intercepts** | **slopes** | **1878.9** | **1917.3** | **-930.4** | **9** | **3** | | **9.99** | **0.018** |  |

*Supplementary Table 1: Model build table, showing comparison to simpler models. RE = random effects, FE = fixed effects, LRT = simulated likelihood ratio test, AIC = Akaike Information Criterion, BIC = Bayes Information Criterion, LL = log-likelihood, df = degrees of freedom. All models are statistically significantly different from the null model.*


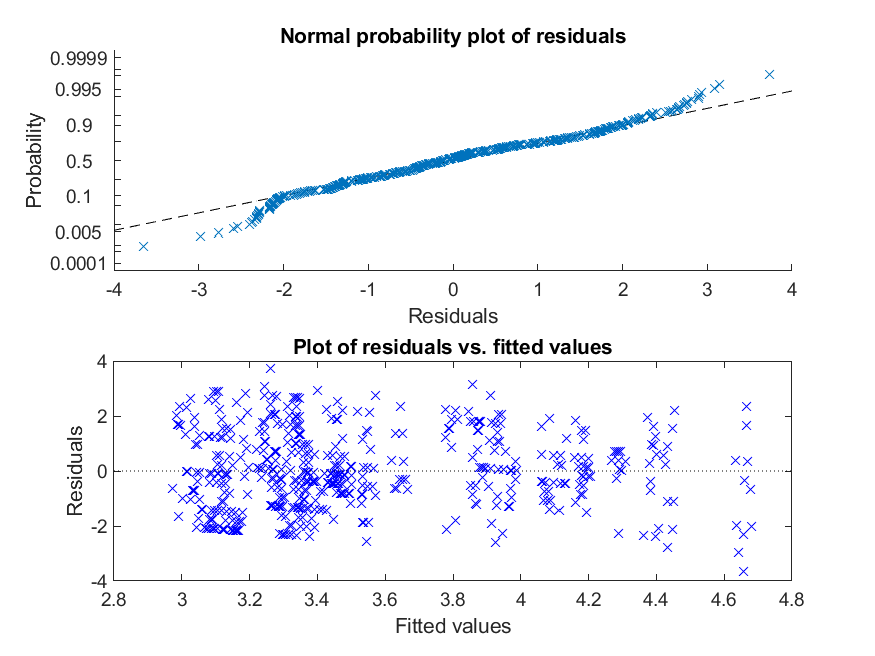


*Supplementary Figure 9: Plots showing distribution of residuals for discomfort ~ SSVEP + model + kurtosis + (1 | image type) + (1 + SSVEP | observer)*

| Fixed Effects | | | | | | |
| --- | --- | --- | --- | --- | --- | --- |
|  | Est/Beta | SE | 95% CI | t | P |  |
| Intercept | 3.179 | 0.237 | 2.71 3.64 | 13.423 | 1.62 x10^-35^ |  |
| SSVEP | -0.004 | 0.017 | -0.04 0.03 | -0.215 | 0.830 |  |
| Total Model | 4.84 x10^-7^ | 1.43x10^-7^ | 2.03x10^-7^ 7.64x10^-7^ | 3.388 | 7.58x10-4 |  |
| Kurtosis | 0.003 | 0.001 | 4.68x10^-4^ 4.96x10^-3^ | 2.374 | 0.018 |  |
|  | | | | | |  |
| Random Effects | | | | | |  |
|  | | | Variance | SD | Correlation |  |
| Image type (intercept) | | | 0.063 | 0.25 |  |  |
| Participant (intercept) | | | 3.60x10^-5^ | 0.006 |  |  |
| Participant (slope) | | | 4.00x10^-6^ | 0.002 | -1 |  |
|  | | | | | |  |
| Model Fit | | | | | |  |
| R2 | | | | Marginal | Conditional |  |
|  | | | | 0.083 | 0.078 |  |
| Key: p-values have been calculated using residual approximations.  Confidence intervals calculated using the Wald method  Model equation: Discomfort ~ SSVEP + model + kurtosis + (1 \| image type) + (1 + SSVEP \| observer) | | | | | |  |

*Supplementary Table 2: Output for the model discomfort ~ SSVEP + model + kurtosis + (1|imagetype) + (1|SSVEP|obs)*

Model equation: discomfort ~ image type + (image type | observer)


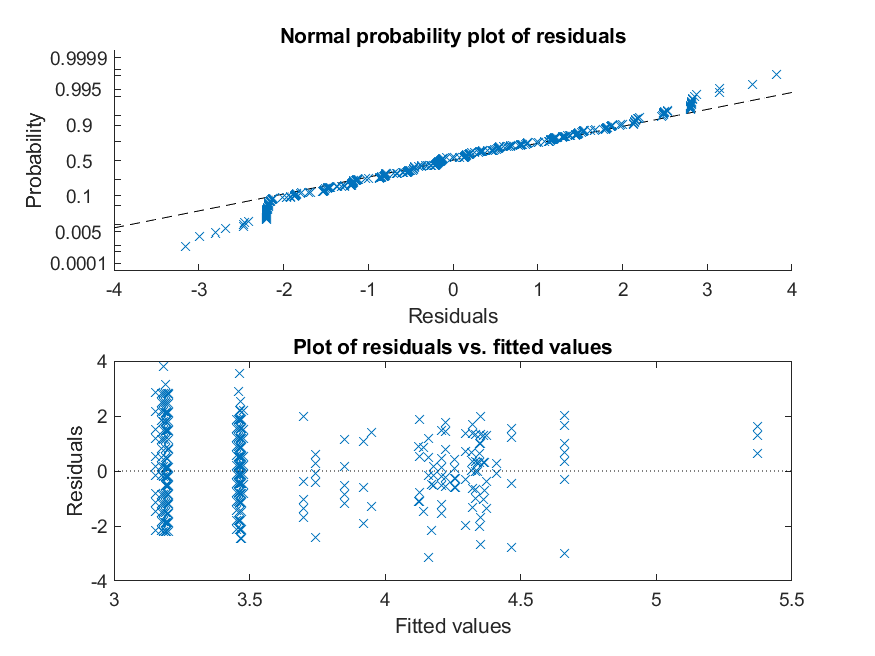


*Supplementary Figure 10: Plots showing distribution of residuals for discomfort ~ image type + (image type | observer)*

This model did not meet the assumptions of the linear mixed effect model due to ordinal structure in the data. As a result, the function “fitmnr” in MATLAB 2023 was used to fit an ordinal regression model. A significant model emerged (X^2^ = (9482) = 49.566, p = 4.45x10^-8^). This showed there to be differences between all the image categories in comparison to the natural images. The output can be seen in Supplementary Table 3. Compared to natural images, artworks were considered to be more comfortable, but all other image categories were considered less comfortable. Please note, this analysis does not incorporate individual as a random effect, as this is not possible with this analysis.

|  | Values | SE | t | p |
| --- | --- | --- | --- | --- |
| Artworks | 0.424 | 0.180 | 2.359 | 0.018 |
| Bump | -0.857 | 0.275 | -3.115 | 0.002 |
| Stripes | -1.039 | 0.276 | -3.761 | 1.695x10^-4^ |
| Riley | -0.942 | 0.398 | -2.369 | 0.018 |

*Supplementary Table 3: table of output for the ordinal regression, SE = standard error.*

| Fixed Effects | | | | | | |
| --- | --- | --- | --- | --- | --- | --- |
|  | Est/Beta | SE | 95% CI | t | P |  |
| Intercept | 3.463 | 0.111 | 3.243 3.683 | 30.957 | 2.508x10^-120^ |  |
| Artworks | -0.278 | 0.144 | -0.561 0.006 | -1.922 | 0.055 |  |
| Bump | 0.767 | 0.222 | 0.330 1.204 | 3.450 | 0.001 |  |
| Stripe | 0.797 | 0.291 | 0.226 1.369 | 2.7409 | 0.006 |  |
| Riley | 0.840 | 0.322 | 0.207 1.472 | 2.609 | 0.009 |  |
|  | | | | | |  |
| Random Effects | | | | | |  |
|  | | | Variance | SD | Correlation |  |
| Participant (intercept) | | | 6.760x10-4 | 0.026 |  |  |
| Artworks (slope) | | | 0.005 | 0.069 | -0.902 |  |
| Bump (slope) | | | 0.034 | 0.183 | 0.259 |  |
| Stripe (slope) | | | 0.411 | 0.641 | -0.403 |  |
| Riley (slope) | | | 0.044 | 0.210 | -0.174 |  |
|  | | |  |  |  |  |
|  | | | | | |  |
| Model Fit | | | | | |  |
| R2 | | | | Marginal | Conditional |  |
|  | | | | 0.083 | 0.078 |  |
| Key: p-values have been calculated using residual approximations.  Confidence intervals calculated using the Wald method  Model equation: Discomfort ~ image type + (1 + image type \| observer) | | | | | |  |

*Supplementary Table 4: Output for the model discomfort ~ image type + (image type | observer)*

Model equation: SSVEP ~ image type + (1 + image type | observer)

Model building process: SSVEP ~ image type + (1 + image type | observer)

| Model specification | Model name | Nested / simpler model | Fixed effects added |  | | Random Effects | | | | Model Fit | | | | | | | | | LRT Test | | | | | |
| --- | --- | --- | --- | --- | --- | --- | --- | --- | --- | --- | --- | --- | --- | --- | --- | --- | --- | --- | --- | --- | --- | --- | --- | --- |
|  | | | | |  | | Subjects | | Items | AIC | BIC | | LL | | df | | df | | | Χ^2^ | | p | |  |
| RE only | Null | - | - |  | | intercepts | | None | | 2502.20 | | 2515.00 | | -1248.10 | | 3 | |  | | |  | |  | |
| FE main effects | Main effects 1 | Null | Image type |  | | intercepts | | None | | 2493.80 | | 2523.60 | | -1239.90 | | 7 | | 4 | | | 16.419 | | 0.003 | |
| FE main effects | Main effects 2 | Null | Image type |  | | intercepts | | slopes | | 2501.20 | | 2590.80 | | -1229.60 | | 21 | | 18 | | | 37.017 | | 0.005 | |

*Supplementary Table 5: Model build table, showing comparison to simpler models. RE = random effects, FE = fixed effects, LRT = simulated likelihood ratio test, AIC = Akaike Information Criterion, BIC = Bayes Information Criterion, LL = log-likelihood, df = degrees of freedom. The model including image type and observer as a random intercept only is the best fitting model according to BIC and AIC, however this is a marginal difference between the intercept only and the inclusion of random slopes.*


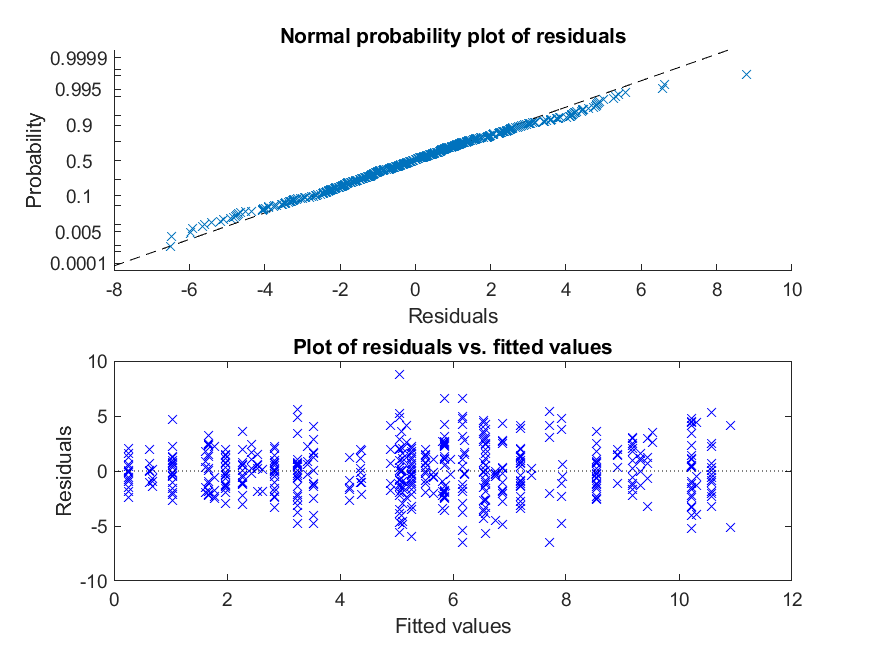


*Supplementary Figure 11: plot showing residual distributions for the model SSVEP ~ image type + (image type | observer)*

| Fixed Effects | | | | | | |
| --- | --- | --- | --- | --- | --- | --- |
|  | Est/Beta | SE | 95% CI | t | P |  |
| Intercept | 5.246 | 0.931 | 3.417 7.074 | 5.637 | 2.837x10^-8^ |  |
| Artworks | 0.011 | 0.323 | -0.623 0.645 | 0.034 | 0.973 |  |
| Bump | -0.458 | 0.419 | -1.281 0.366 | -1.092 | 0.275 |  |
| Stripes | -0.054 | 0.533 | -1.102 0.994 | -0.101 | 0.919 |  |
| Riley | 1.990 | 0.729 | 0.559 3.421 | 2.731 | 0.006 |  |
|  | | | | | |  |
| Random Effects | | | | | |  |
|  | | | Variance | SD | Correlation |  |
| Participant (intercept) | | | 9.133 | 3.022 |  |  |
| Artworks | | | 0.510 | 0.714 | -0.515 |  |
| Bump | | | 0.453 | 0.673 | -0.804 |  |
| Stripe | | | 1.651 | 1.285 | -0.360 |  |
| Riley | | | 2.726 | 1.651 | -0.376 |  |
|  | | | | | |  |
| Model Fit | | | | | |  |
| R^2^ | | | | Marginal | Conditional |  |
|  | | | | 0.601 | 0.598 |  |
| Key: p-values have been calculated using residual approximations.  Confidence intervals calculated using the Wald method  Model equation: SSVEP ~ image type + (image type \| observer) | | | | | |  |

*Supplementary Table 6: Output for the model SSVEP ~ image type + (image type | observer)*

Model equation: discomfort ~ 1 + sf + sf^2^ + image type:(sf^2^) – image type:(sf) + (1 + sf + image type | observer)

Model building process: discomfort ~ 1 + sf + sf^2^ + image type:(sf^2^) – image type:(sf) + (1 + sf + image type | observer)

| Model specification | Model name | Nested / simpler model | Fixed effects added |  | | Random Effects | | | | Model Fit | | | | | | | | | LRT Test | | | |
| --- | --- | --- | --- | --- | --- | --- | --- | --- | --- | --- | --- | --- | --- | --- | --- | --- | --- | --- | --- | --- | --- | --- |
|  | | | | |  | | Subjects | | Items | | AIC | | BIC | | LL | | df | | df | | Χ^2^ | |
| RE only | Null | - | - |  | | intercepts | | slopes | | 385.28 | | 406.88 | | -184.64 | | 8 | |  | |  | |  |
| FE main effects | Main effects 1 | Null | SF |  | | intercepts | | slopes | | 386.38 | | 410.68 | | -184.19 | | 9 | | 1 | | 0.899 | |  |
| FE main effects | Main effects 2 | Null | SF^2^ |  | | intercepts | | slopes | | 386.92 | | 411.22 | | -184.46 | | 9 | | 1 | | 0.358 | |  |
| FE main effects | Main effects 3 | Null | SF + SF^2^ |  | | intercepts | | slopes | | 385.54 | | 412.54 | | -182.77 | | 10 | | 2 | | 3.736 | |  |
| **FE main effects and interaction** | **Interaction** | **Main effects 3** | **SF + SF^2^ + SF * SF^2^** |  | | **intercepts** | | **slopes** | | **379.29** | | **409.00** | | **-178.65** | | **11** | | **1** | | **8.249** | |  |

*Supplementary Table 7: Model build table, showing comparison to simpler models. RE = random effects, FE = fixed effects, LRT = simulated likelihood ratio test, AIC = Akaike Information Criterion, BIC = Bayes Information Criterion, LL = log-likelihood, df = degrees of freedom, SF = spatial frequency. The best fitting model included all terms.*

Model equation: discomfort ~ 1 + sf + sf^2^ + image type:(sf^2^) – image type:(sf) + (1 + sf + image type | observer)


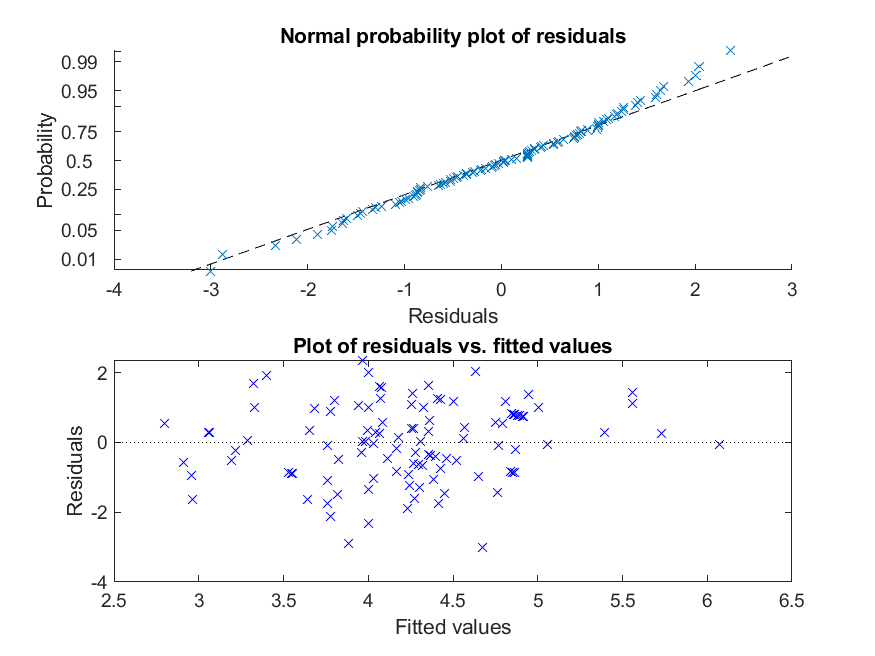


*Supplementary Figure 12: Plot showing distribution of residuals for the model discomfort ~ 1 + sf + sf2 + image type:(sf2) – image type:(sf) + (1 + sf + image type | observer)*

| Fixed Effects | | | | | | |
| --- | --- | --- | --- | --- | --- | --- |
|  | Est/Beta | SE | 95% CI | t | P |  |
| Intercept | 5.304 | 0.521 | 4.271 6.337 | 10.178 | 2.21x10^-17^ |  |
| SF | -0.828 | 0.398 | -1.617 -0.040 | -2.082 | 0.040 |  |
| SF^2 | 0.149 | 0.066 | 0.019 0.279 | 2.266 | 0.025 |  |
| Image type*SF | -0.064 | 0.021 | -0.106 -0.023 | -3.090 | 0.003 |  |
|  | | | | | |  |
| Random Effects | | | | | |  |
|  | | | Variance | SD | Correlation |  |
| Participant (intercept) | | | 0.341 | 0.584 |  |  |
| Image type | | | 1.323 | 1.150 | -1 |  |
| Spatial frequency | | | 0.011 | 0.104 | -1 |  |
|  | | | | | |  |
| Model Fit | | | | | |  |
| R^2^ | | | | Marginal | Conditional |  |
|  | | | | 0.244 | 0.222 |  |
| Key: p-values have been calculated using residual approximations.  Confidence intervals calculated using the Wald method  Model equation: discomfort ~ 1 + sf + sf^2^ + image type:(sf^2^) – image type:(sf) + (1 + sf + image type \| observer) | | | | | |  |

*Supplementary Table 8: Output for the model discomfort ~ 1 + sf + sf2 + image type:(sf2) – image type:(sf) + (1 + sf + image type | observer)*

Model equation: SSVEP ~ image type + sf + (1 + sf + image type | observer)

Model building process: SSVEP ~ image type + sf + (1 + sf + image type | observer)

| Model specification | Model name | Nested / simpler model | Fixed effects added |  | | Random Effects | | | | Model Fit | | | | | | | | | LRT Test | | | | | |
| --- | --- | --- | --- | --- | --- | --- | --- | --- | --- | --- | --- | --- | --- | --- | --- | --- | --- | --- | --- | --- | --- | --- | --- | --- |
|  | | | | |  | | Subjects | | Items | | AIC | | BIC | | LL | | df | | df | | Χ^2^ | | p | |
| RE only | Null1 | - | - |  | | intercepts | | Slopes | | 529.61 | | 551.22 | | -256.81 | | 8 | |  | |  | |  | |  |
| FE main effects | Main effects 1 | Null | Image type |  | | intercepts | | Slopes | | 531.19 | | 555.49 | | -256.60 | | 9 | | 1 | | 0.424 | | 0.515 | |  |
| **FE main effects** | **Main effects 2** | **Null** | **SF** |  | | **intercepts** | | **Slopes** | | **527.42** | | **551.72** | | **254.71** | | **9** | | **1** | | **4.196** | | **0.041** | |  |
| FE main effects | Main effects 3 | Null | Image type + SF |  | | intercepts | | slopes | | 528.95 | | 555.96 | | -254.48 | | 10 | | 2 | | 4.664 | | 0.097 | |  |

*Supplementary Table 9: Model build table, showing comparison to simpler models. RE = random effects, FE = fixed effects, LRT = simulated likelihood ratio test, AIC = Akaike Information Criterion, BIC = Bayes Information Criterion, LL = log-likelihood, df = degrees of freedom.*


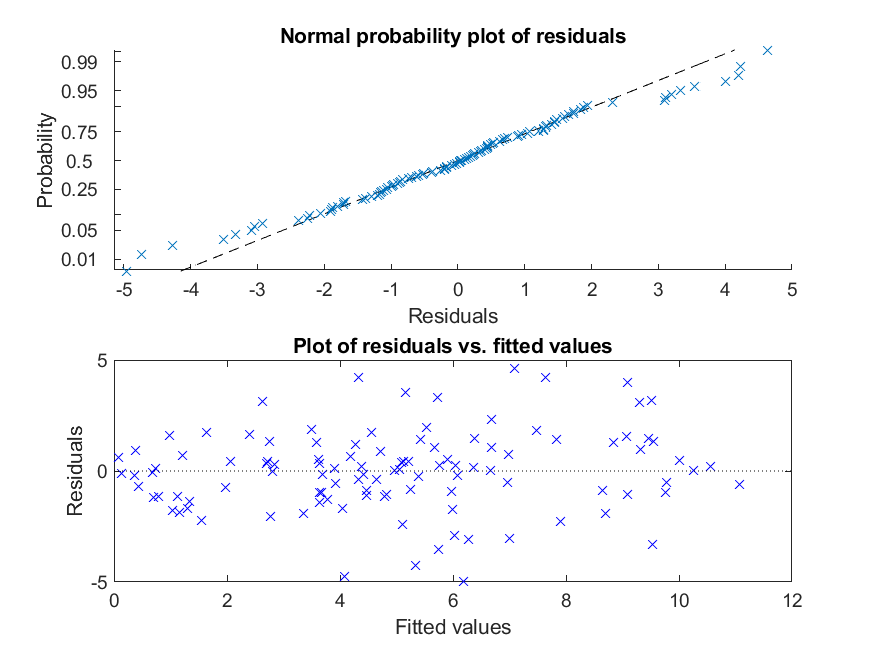


*Supplementary Figure 13: Plot showing distribution of residuals for the model SSVEP ~ image type + sf + (1 + sf + image type | observer)*

| Fixed Effects | | | | | | |
| --- | --- | --- | --- | --- | --- | --- |
|  | Est/Beta | SE | 95% CI | t | P |  |
| Intercept | 3.457 | 0.958 | 1.559 5.356 | 3.611 | 4.664x10^-4^ |  |
| Image type | 0.404 | 0.429 | -0.446 1.254 | 0.941 | 0.349 |  |
| Spatial frequency | 0.443 | 0.151 | 0.143 0.744 | 2.925 | 0.004 |  |
|  | | | | | |  |
| Random Effects | | | | | |  |
|  | | | Variance | SD | Correlation |  |
| Participant (intercept) | | | 6.796 | 2.607 |  |  |
| Image type (slope) | | | 2.176 | 1.475 | -524 |  |
| Spatial frequency (slope) | | | 0.225 | 0.474 | -529 |  |
|  | | | | | |  |
| Model Fit | | | | | |  |
| R^2^ | | | | Marginal | Conditional |  |
|  | | | | 0.596 | 0.588 |  |
| Key: p-values have been calculated using residual approximations.  Confidence intervals calculated using the Wald method  Model equation: SSVEP ~ image type + sf + (1 + sf + image type \| observer) | | | | | |  |

*Supplementary Table 10: Output for the model SSVEP ~ image type + sf + (1 + sf + image type | observer)*

Model equation: discomfort ~ PCA1 + PCA2 + PCA3 + (1|obs)

This did not meet the assumptions of the linear mixed effect model due to clustering in the residuals.


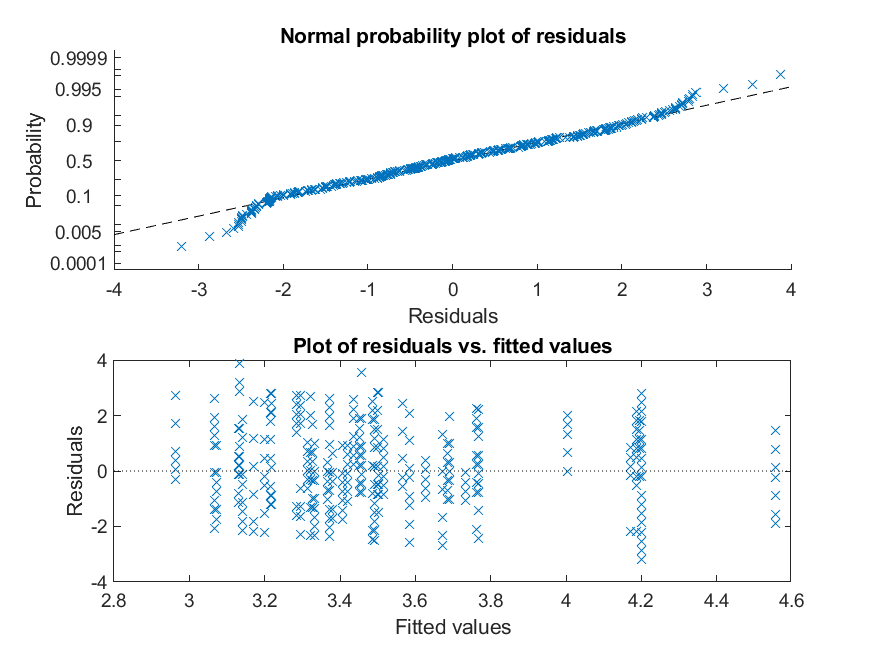


*Supplementary Figure 14: Plot showing distribution of the residuals for the model discomfort ~ PCA1 + PCA2 + PCA3 + (1 | observer)*

As a result, ordinal regression was used to determine the relationship between discomfort and the three principal components. A significant model emerged (X^2^ (9438) = 32.448, p = 4.21x10^-7^). Supplementary Table 11 shows the output. PCA1 and PCA3 were statistically significant.

|  | Value | SE | t | p |
| --- | --- | --- | --- | --- |
| PCA1 | 0.146 | 0.0354 | 4.129 | 3.645x10^-5^ |
| PCA2 | 0.120 | 0.062 | 1.941 | 0.052 |
| PCA3 | 0.283 | 0.081 | 3.511 | 4.472x10^-4^ |

*Supplementary Table 11: table of output for the ordinal regression, SE = standard error.*

| Fixed Effects | | | | | | |
| --- | --- | --- | --- | --- | --- | --- |
|  | Est/Beta | SE | 95% CI | t | P |  |
| Intercept | 3.534 | 0.062 | 3.413 3.656 | 57.108 | 3.782x10^-227^ |  |
| PCA1 | -0.119 | 0.029 | -0.175 -0.063 | -4.156 | 3.786x10^-5^ |  |
| PCA2 | -0.084 | 0.050 | -0.184 0.015 | -1.671 | 0.095 |  |
| PCA3 | -0.243 | 0.065 | -0.371 -0.115 | -3.724 | 2.176x10^-4^ |  |
|  | | | | | |  |
| Random Effects | | | | | |  |
|  | | | Variance | SD | Correlation |  |
| Participant (intercept) | | | 6.498x10^-15^ | 8.061x10^-8^ |  |  |
|  | | | | | |  |
| Model Fit | | | | | |  |
| R^2^ | | | | Marginal | Conditional |  |
|  | | | | 0.060 | 0.055 |  |
| Key: p-values have been calculated using residual approximations.  Confidence intervals calculated using the Wald method  Model equation: discomfort ~ PCA1 + PCA2 + PCA3 + (1\|observer) | | | | | |  |

*Supplementary Table 12: Output for the model discomfort ~ PCA1 + PCA2 + PCA3 + (1|observer)*

Model Equation: SSVEP ~ PCA1 + PCA2


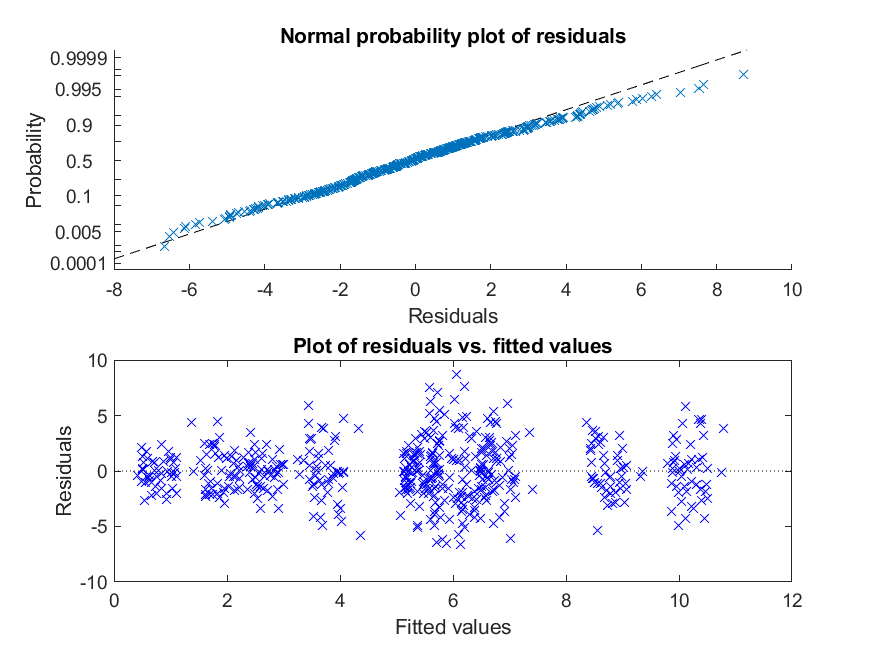


*Supplementary Figure 15: Plot showing distribution of residuals for the model SSVEP ~ PCA1 + PCA2 + (1 | observer).*

When the PCA3 was included, this clustering disappeared. See Supplementary Figure 16. Therefore all 3 PCA components were included in the model.

Model equation: SSVEP ~ PCA1 + PCA2 + PCA3 + (1 | observer)

Model building process: SSVEP ~ PCA1 + PCA2 + PCA3 + (1 | observer)

| Model specification | Model name | Nested / simpler model | Fixed effects added |  | | Random Effects | | | | Model Fit | | | | | | LRT Test | | |
| --- | --- | --- | --- | --- | --- | --- | --- | --- | --- | --- | --- | --- | --- | --- | --- | --- | --- | --- |
|  | | | | |  | | Subjects | | Items | | AIC | BIC | LL | df | df | | Χ^2^ |  |
| RE only | Null | - | - |  | | intercepts | | slopes | | 2502.20 | | 2515.00 | -1248.10 | 3 |  | |  |  |
| FE main effects | Main effects 1 | Null | PCA1 |  | | intercepts | | Intercepts | | 2503.30 | | 2520.40 | -1247.70 | 4 | 1 | | 0.857 |  |
| FE main effects | Main effects 2 | Null | PCA2 |  | | intercepts | | intercepts | | 2500.10 | | 2517.20 | -1246.00 | 4 | 1 | | 4.107 |  |
| FE main effects | Main effects 3 | Null | PCA1 + PCA2 |  | | intercepts | | intercepts | | 2501.20 | | 2522.60 | -1245.60 | 5 | 2 | | 4.971 |  |
| FE main effects | Main effects 4 | Null | PCA1 + PCA2 + PCA3 |  | | intercepts | | intercepts | | 2473.50 | | 2499.10 | -1230.80 | 6 | 3 | | 34.657 |  |

*Supplementary Table 13: Model build table, showing comparison to simpler models. RE = random effects, FE = fixed effects, LRT = simulated likelihood ratio test, AIC = Akaike Information Criterion, BIC = Bayes Information Criterion, LL = log-likelihood, df = degrees of freedom. Main effects model 4 has the lowest AIC and BIC values and so the decision was to include all three principal components.*


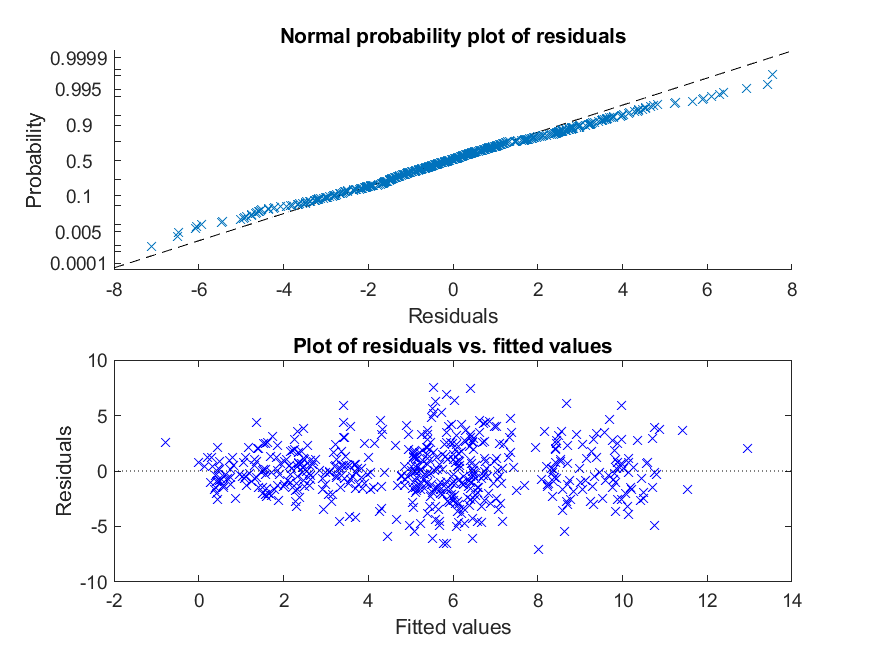


*Supplementary Figure 16: Plot showing distribution of residuals for the model SSVEP ~ PCA1 + PCA2 + PCA3 + (1 | observer)*

| Fixed Effects | | | | | | |
| --- | --- | --- | --- | --- | --- | --- |
|  | Est/Beta | SE | 95% CI | t | P |  |
| Intercept | 5.280 | 0.830 | 3.649 6.911 | 6.360 | 4.406x10^-10^ |  |
| PCA1 | 0.046 | 0.048 | -0.048 0.140 | 0.957 | 0.339 |  |
| PCA2 | 0.177 | 0.085 | 0.011 0.343 | 2.091 | 0.037 |  |
| PCA3 | -0.605 | 0.101 | -0.820 -0.390 | -5.528 | 5.125x10^-8^ |  |
|  | | | | | |  |
| Random Effects | | | | | |  |
|  | | | Variance | SD | Correlation |  |
| Participant (intercept) | | | 7.464 | 2.732 |  |  |
|  | | | | | |  |
| Model Fit | | | | | |  |
| R^2^ | | | | Marginal | Conditional |  |
|  | | | | 0.581 | 0.579 |  |
| Key: p-values have been calculated using residual approximations.  Confidence intervals calculated using the Wald method  Model equation: SSVEP ~ PCA1 + PCA2 + PCA3 + (1 \| observer) | | | | | |  |

*Supplementary Table 14: Output for the model SSVEP ~ PCA1 + PCA2 + PCA3 + (1 | observer)*

Model Equation: discomfort ~ RMS + (1 | image type) + (1 + RMS | observer)


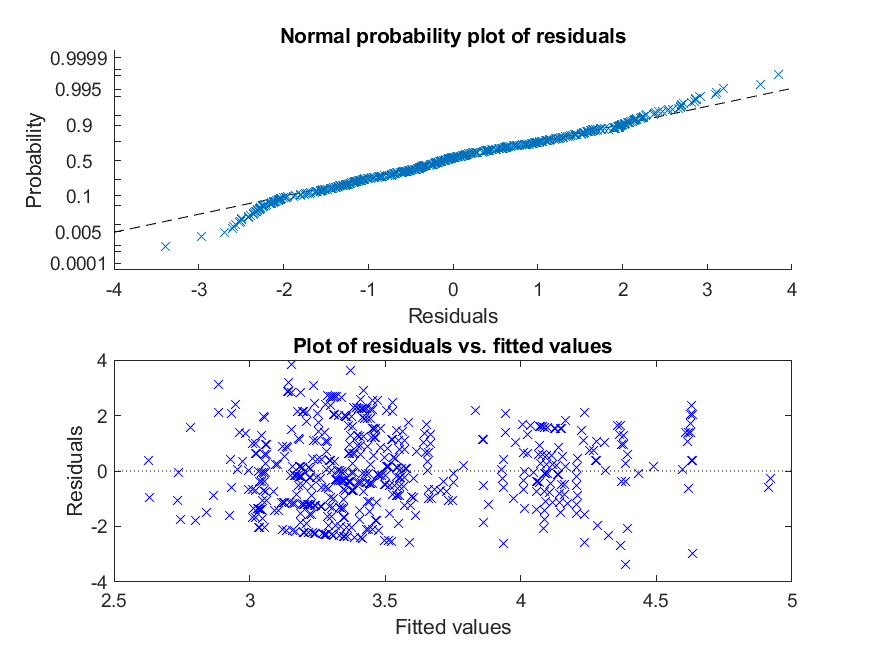


*Supplementary Figure 17: Plot showing distribution of residuals for the model discomfort ~ RMS + (1 | image type) + (1 + RMS | observer)*.

| Fixed Effects | | | | | | |
| --- | --- | --- | --- | --- | --- | --- |
|  | Est/Beta | SE | 95% CI | t | P |  |
| Intercept | 3.113 | 0.335 | 2.455 3.772 | 9.286 | 4.211x10^-19^ |  |
| RMS | 3.322 | 1.307 | 0.755 5.889 | 2.542 | 0.11 |  |
|  | | | | | |  |
| Random Effects | | | | | |  |
|  | | | Variance | SD | Correlation |  |
| Participant (intercept) | | | 0.092 | 0.304 |  |  |
| Participant (RMS slope) | | | 3.606 | 1.899 | -1 |  |
| Image type (intercept) | | | 0.160 | 0.400 |  |  |
|  | | | | | |  |
| Model Fit | | | | | |  |
| R^2^ | | | | Marginal | Conditional |  |
|  | | | | 0.092 | 0.091 |  |
| Key: p-values have been calculated using residual approximations.  Confidence intervals calculated using the Wald method  Model equation: discomfort ~ rms + (1\|imagetype) + (1+rms\|obs) | | | | | |  |

*Supplementary Table 15: Output for the model discomfort ~ rms + (1|imagetype) + (1+rms|obs)*

Model Equation: discomfort ~ effective + (1 | image type) + (1 + effective | observer)


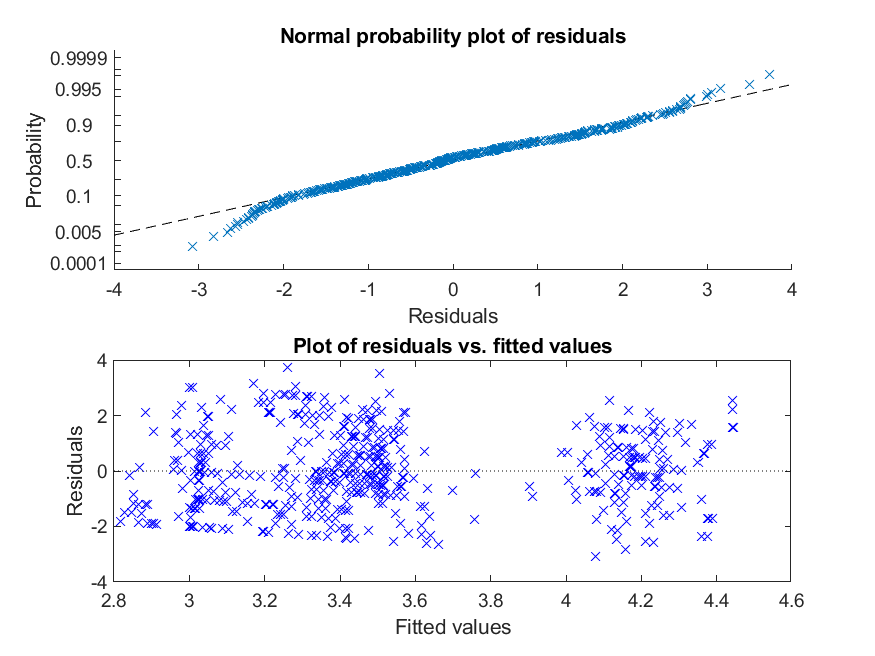


*Supplementary Figure 18: Plot showing distribution of residuals for the model discomfort ~ effective + (1 | image type) + (1 + effective | observer).*

| Fixed Effects | | | | | | |
| --- | --- | --- | --- | --- | --- | --- |
|  | Est/Beta | SE | 95% CI | t | p |  |
| Intercept | 3.661 | 0.298 | 3.075 4.248 | 12.272 | 1.283x10^-30^ |  |
| Effective | 0.022 | 0.010 | 0.002 0.041 | 0.010 | 0.029 |  |
|  |  |  |  |  |  |  |
|  | | | | | |  |
| Random Effects | | | | | |  |
|  | | | Variance | SD | Correlation |  |
| Participant (intercept) | | | 0.040 | 0.199 |  |  |
| Participant (slope) | | | 1.96x10^-4^ | 0.014 | -1 |  |
| Image type (intercept) | | | 0.359 | 0.599 |  |  |
|  | | | | | |  |
| Model Fit | | | | | |  |
| R^2^ | | | | Marginal | Conditional |  |
|  | | | | 0.091 | 0.089 |  |
| Key: p-values have been calculated using residual approximations.  Confidence intervals calculated using the Wald method  Model equation: discomfort ~ effective + (1 \| image type) + (1 + effective \| observer) | | | | | |  |

*Supplementary Table 16: Output for the model discomfort ~ effective + (1 | image type) + (1 + effective | observer)*

**3) Eye Movements**

One way of assessing eye movement is to assess the number of trials rejected due to artefact as many of these will likely be due to eye movement. As observer 8 had on average of 46% trials rejected across all image categories, this observer was not included in the final analysis. For the remaining 11 observers, the mean number of rejected epochs was estimated as: artworks 1.07 (SD = 2.56), the work of Bridget Riley 1.23, (SD = 2.29), bump stimuli 0.73 (SD = 2.35), stripe stimuli 1.40 (SD = 2.61), natural images 0.89 (SD = 2.09).

Eye channels were estimated by the method of Jia and Tyler, (2019). The vertical eye channel was estimated by taking the difference of the sub-orbital and supra-orbital channels on the left side of the participant. The horizontal eye channel was estimated by taking the difference of the channels positioned on the two outer canthi.

Horizontal eye channel

Supplementary Figure 19 shows the spectra for the horizontal eye channel response, there is no discernible peak at 5Hz. There was no relationship between the eye channel response and the discomfort judgements (-0.002,± 0.02 SE, p = 0.89, CI =[-0.04 0.03]). There was no relationship between eye channel response and spatial frequency content of the stripe and bump stimuli (-0.09, ± 0.13 SE, p = 0.51, CI = [-0.35, 0.17]). There was no relationship between the horizonal eye channel response and the PCA components, PCA1 (-0.01, ± 0.05 SE, p = 0.86, CI = [-0.11 0.09]), PCA2 (0.08, ± 0.09 SE, p = 0.41, CI =[-0.11, 0.26]).


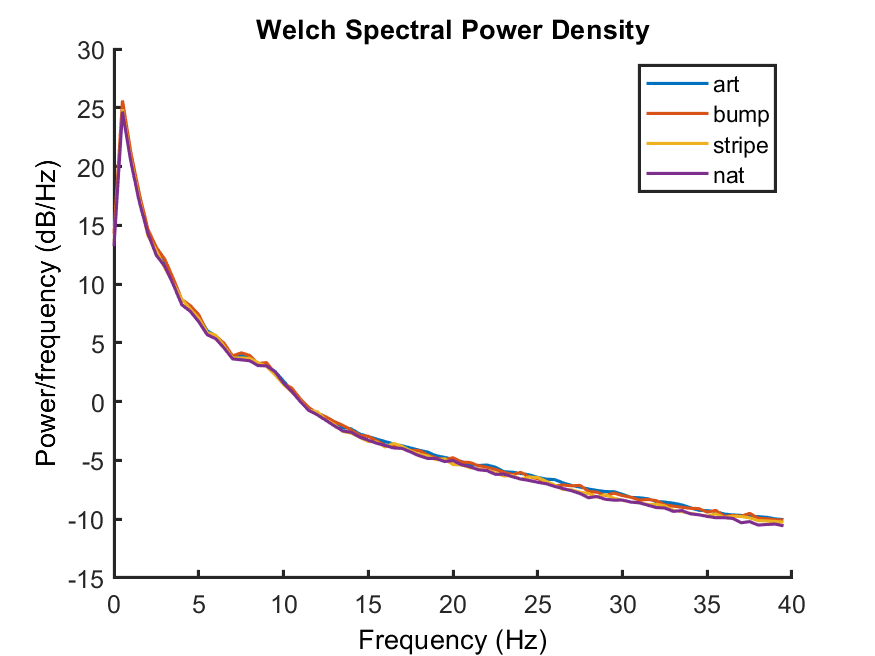


*Supplementary Figure 19: Spectrum showing horizontal eye channel response.*

| Fixed Effects | | | | | | |
| --- | --- | --- | --- | --- | --- | --- |
|  | Est/Beta | SE | 95% CI | t | p |  |
| Intercept | 3.864 | 0.251 | 3.371 4.356 | 15.404 | 1.841x10^-44^ |  |
| SSVEP | -0.002 | 0.018 | -0.037 0.032 | -0.140 | 0.889 |  |
|  | | | | | |  |
| Random Effects | | | | | |  |
|  | | | Variance | SD | Correlation |  |
| Participant (intercept) | | | 7.285x10^-22^ | 2.699x10^-11^ |  |  |
| SSVEP (slope) | | | 3.629x10^-24^ | 1.905x10^-12^ | -0.889 |  |
|  | | | | | |  |
| Model Fit | | | | | |  |
| R^2^ | | | | Marginal | Conditional |  |
|  | | | | 0.072 | 0.070 |  |
| Key: p-values have been calculated using residual approximations.  Confidence intervals calculated using the Wald method  Model equation: discomfort ~ SSVEP + (1\|image type) + (1 + SSVEP \| observer) | | | | | |  |

*Supplementary Table 17: Output of the model discomfort ~ SSVEP + (1|image type) + (1 + SSVEP | observer) for the horizontal eye channel*


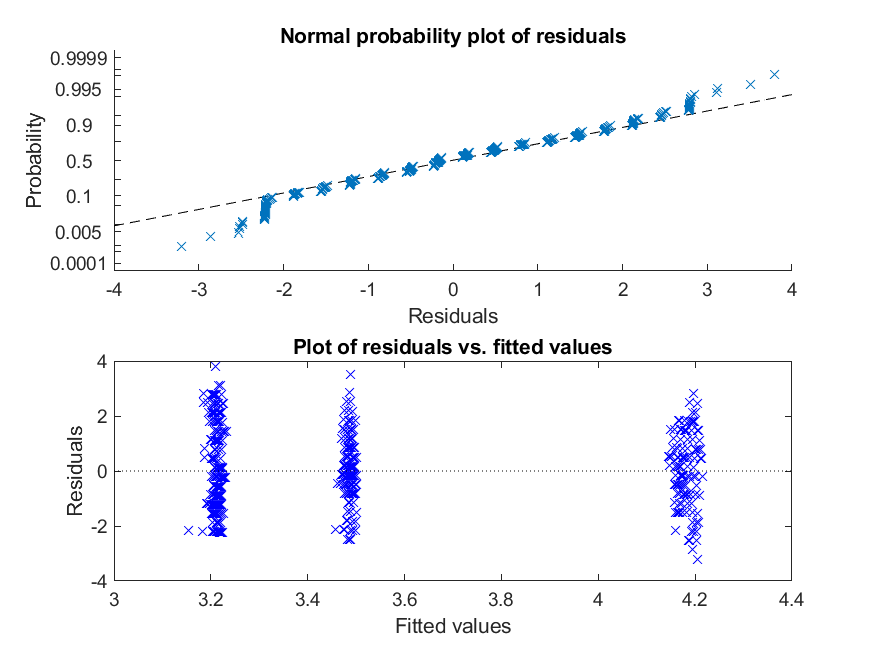


*Supplementary Figure 20: assumptions of the linear mixed effect model for the* *discomfort ~ SSVEP + (1|image type) + (1 + SSVEP | observer) for the horizontal eye channel*

As this did not meet the assumptions of the linear mixed effect model, a more conservative ordinal regression was conducted to predict discomfort from the horizontal eye channel. The model was not significant (X^2^ (9485) = 0.015, p = 0.904). This showed there to be no relationship between the horizontal eye channel response and the discomfort judgements (0.003 ± 0.022 SE, t = 0.127, p = 0.899)


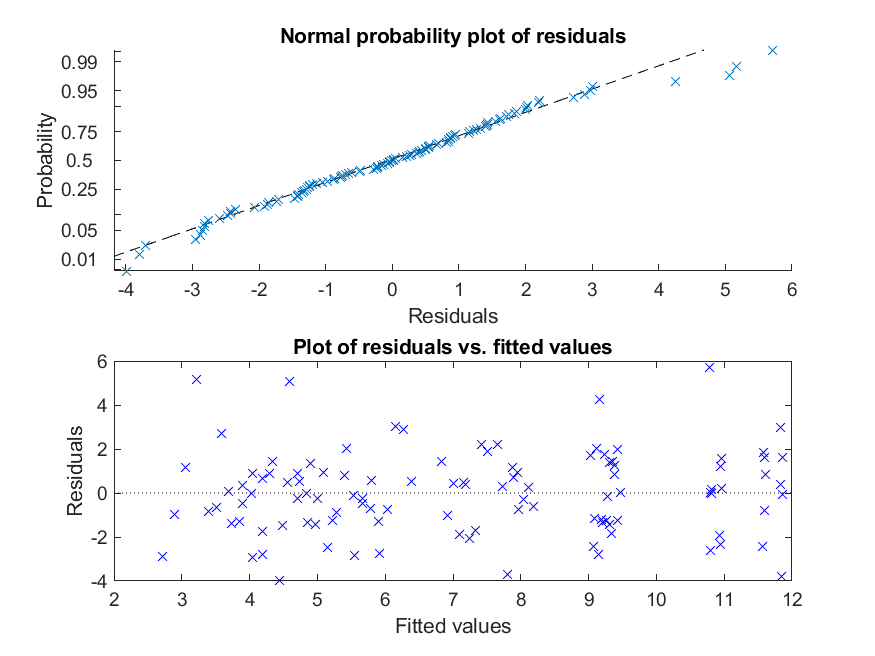


*Supplementary Figure 21: Assumptions of the linear mixed effect model for SSVEP ~ image type + SF + (1+ image type + SF | observer) for the horizontal eye channel*

| Fixed Effects | | | | | | |
| --- | --- | --- | --- | --- | --- | --- |
|  | Est/Beta | SE | 95% CI | t | p |  |
| Intercept | 7.653 | 0.891 | 5.947 9.359 | 8.891 | 1.635x10^-14^ |  |
| Image type | -0.317 | 0.386 | -1.082 0.448 | -0.821 | 0.413 |  |
| SF | -0.087 | 0.133 | -0.350 0.176 | -0.656 | 0.513 |  |
|  |  |  |  |  |  |  |
|  | | | | | |  |
| Random Effects | | | | | |  |
|  | | | Variance | SD | Correlation |  |
| Participant (intercept) | | | 5.684 | 2.384 |  |  |
| SSVEP (slope) | | | 0.122 | 0.349 |  |  |
| SF (slope) | | | 0.004 | 0.059 | 1 |  |
|  | | | | | |  |
| Model Fit | | | | | |  |
| R^2^ | | | | Marginal | Conditional |  |
|  | | | | 0.677 | 0.671 |  |
| Key: p-values have been calculated using residual approximations.  Confidence intervals calculated using the Wald method  Model equation: SSVEP ~ image type + SF + (1+ image type + SF \| observer) | | | | | |  |

*Supplementary Table 18: Output of the model SSVEP ~ image type + SF + (1+ image type + SF | observer) for the horizontal eye channel*

| Fixed Effects | | | | | | |
| --- | --- | --- | --- | --- | --- | --- |
|  | Est/Beta | SE | 95% CI | t | p |  |
| Intercept | 7.129 | 0.704 | 5.746 8.512 | 10.128 | 3.868x10^-22^ |  |
| PCA1 | -0.001 | 0.053 | -0.114 0.095 | -0.180 | 0.858 |  |
| PCA2 | 0.078 | 0.094 | -0.106 0.262 | 0.832 | 0.406 |  |
| PCA3 | -0.107 | 0.121 | -0.344 0.131 | -0.880 | 0.379 |  |
|  | | | | | |  |
| Random Effects | | | | | |  |
|  | | | Variance | SD | Correlation |  |
| Participant (intercept) | | | 5.304 | 2.303 |  |  |
|  | | | | | |  |
| Model Fit | | | | | |  |
| R^2^ | | | | Marginal | Conditional |  |
|  | | | | 0.432 | 0.429 |  |
| Key: p-values have been calculated using residual approximations.  Confidence intervals calculated using the Wald method  Model equation: SSVEP ~ PCA1 + PCA2 + PCA3 + (1 \| observer) | | | | | |  |

*Supplementary Table 19: Output of the model SSVEP ~ PCA1 + PCA2 + PCA3 + (1 | observer) for the horizontal eye channel*


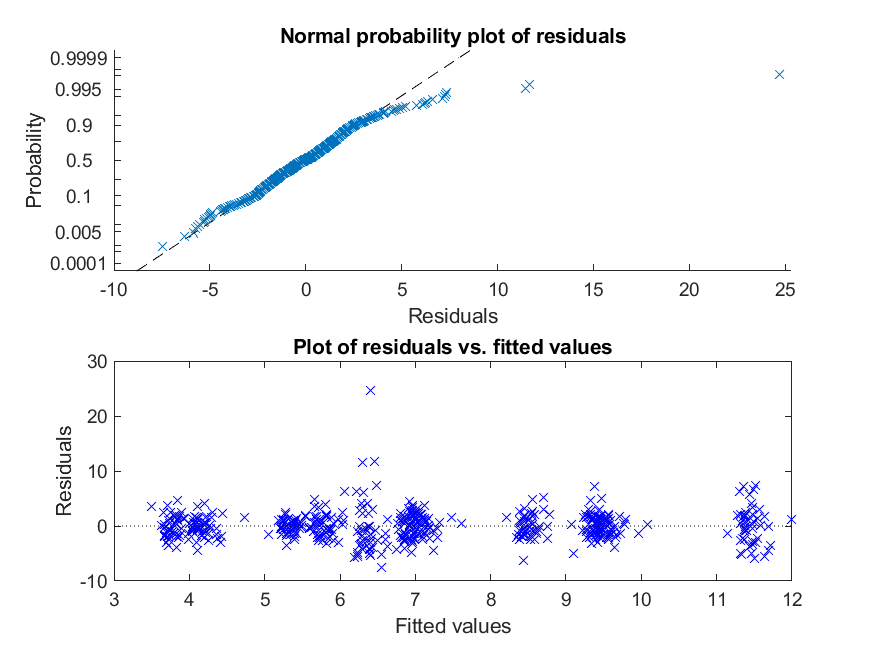


*Supplementary Figure 22: Assumptions of the linear mixed effect model for SSVEP ~ PCA1 + PCA2 + PCA3 + (1 | observer) for the horizontal eye channel*

Vertical eye channel

Supplementary Figure 20 shows the spectra for the vertical eye channel response, there is again no discernible peak at 5Hz. There was no relationship between the vertical eye channel response and the discomfort judgements (-0.01, ± 0.01 SE, p = 0.38, CI =[-0.03, 0.01]). There was no relationship between eye channel response and spatial frequency content of the stripe and bump stimuli (0.23, ± 0.18 SE, p = 0.19 CI = [-0.12, 0.60]). There was no relationship between the horizonal eye channel response and the PCA components, PCA1 (0.08, ± 0.06 SE, p = 0.14, CI = [-0.03, 0.19]), PCA2 (3.72x10-5, ± 0.10 SE, p = 0.99, CI =[-0.20, 0.20]).


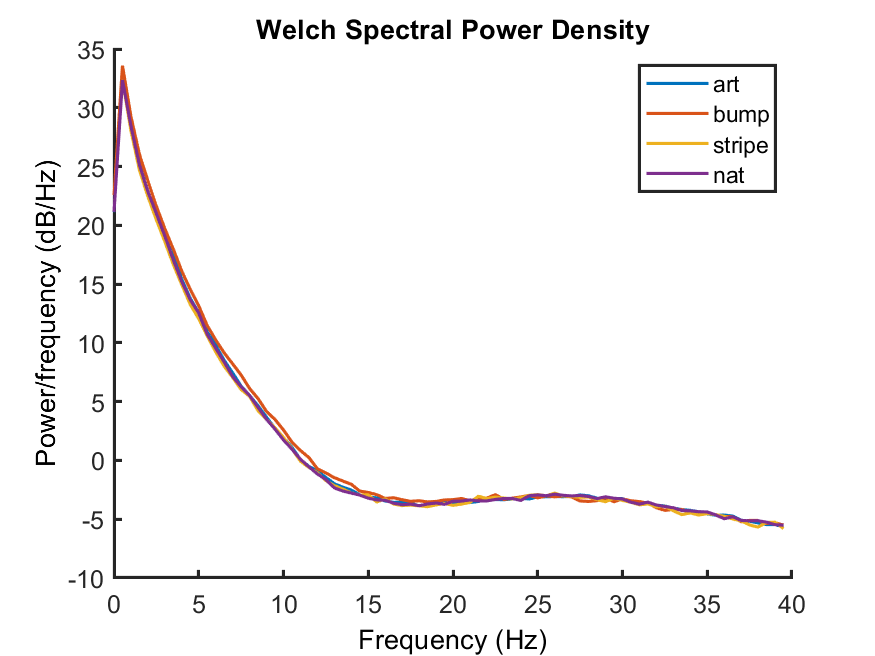


*Supplementary Figure 23: Spectrum showing vertical eye channel response.*

| Fixed Effects | | | | | | |
| --- | --- | --- | --- | --- | --- | --- |
|  | Est/Beta | SE | 95% CI | t | p |  |
| Intercept | 3.975 | 0.262 | 3.460 4.489 | 15.178 | 2.031x10-43 |  |
| SSVEP | -0.010 | 0.012 | -0.033 0.013 | -0.877 | 0.381 |  |
|  | | | | | |  |
| Random Effects | | | | | |  |
|  | | | Variance | SD | Correlation |  |
| Participant (intercept) | | | 6.355x10^-18^ | 2.521x10^-9^ |  |  |
| SSVEP (slope) | | | 1.714x10^-20^ | 1.309x10^-10^ | -0.999 |  |
|  | | | | | |  |
| Model Fit | | | | | |  |
| R^2^ | | | | Marginal | Conditional |  |
|  | | | | 0.074 | 0.072 |  |
| Key: p-values have been calculated using residual approximations.  Confidence intervals calculated using the Wald method  Model equation: discomfort ~ SSVEP + (1\|image type) + (1 + SSVEP \| observer) | | | | | |  |

*Supplementary Table 20: Output for the model discomfort ~ SSVEP + (1|image type) + (1 + SSVEP | observer) for the vertical eye channels*


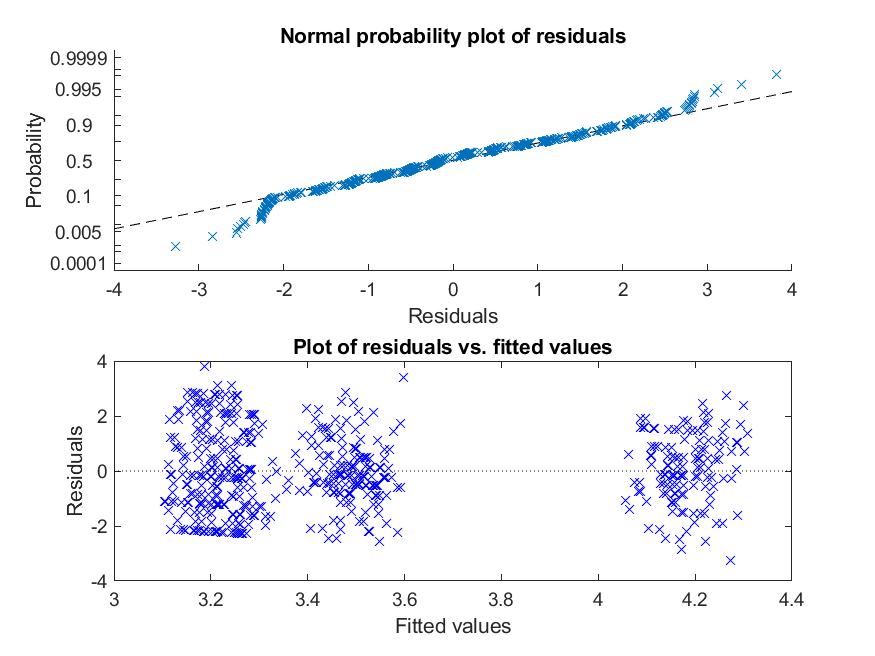


*Supplementary Figure 24: Assumptions of the linear mixed effect model for* *discomfort ~ SSVEP + (1|image type) + (1 + SSVEP | observer) for the vertical eye channels*

As this did not meet the assumptions of the linear mixed effect model, a more conservative ordinal regression was conducted to predict discomfort from the horizontal eye channel. The model was not significant (X^2^ (9485) = 0.461, p = 0.497). This showed there to be no relationship between the horizontal eye channel response and the discomfort judgements (0.010 ± 0.014 SE, t = 0.678, p = 0.498)


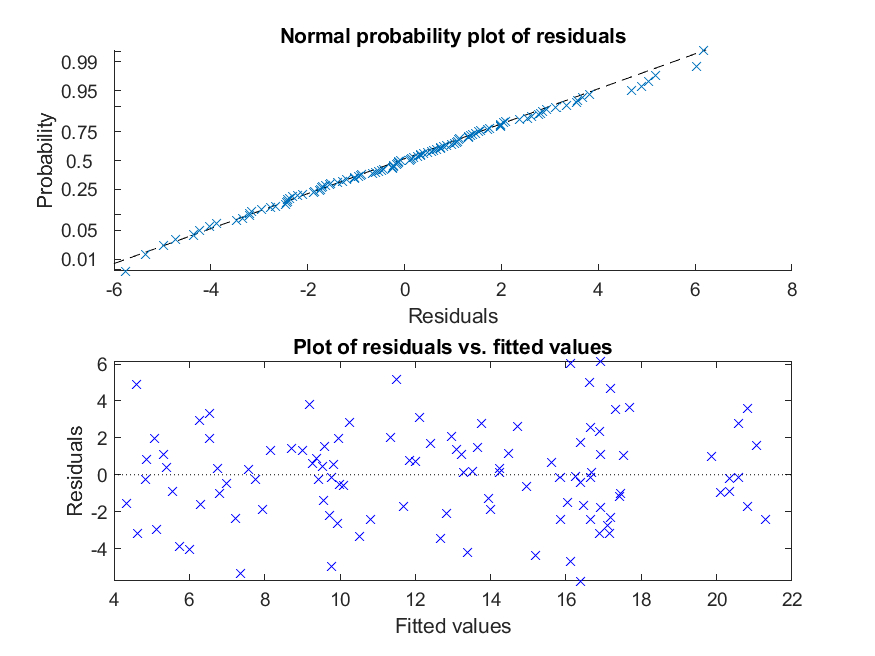


*Supplementary Figure 25: Assumptions of the linear mixed effect model for* *SSVEP ~ image type + SF + (1+ image type + SF | observer) for the vertical eye channel*

| Fixed Effects | | | | | | |
| --- | --- | --- | --- | --- | --- | --- |
|  | Est/Beta | SE | 95% CI | t | p |  |
| Intercept | 12.428 | 1.518 | 9.420 15.437 | 8.190 | 6.030x10^-13^ |  |
| Image type | -1.095 | 0.568 | -2.220 0.031 | -1.928 | 0.056 |  |
| SF | 0.239 | 0.181 | -0.118 0.597 | 1.327 | 0.187 |  |
|  |  |  |  |  |  |  |
|  | | | | | |  |
| Random Effects | | | | | |  |
|  | | | Variance | SD | Correlation |  |
| Participant (intercept) | | | 20.757 | 4.556 |  |  |
| SSVEP (slope) | | | 0.726 | 0.852 | -0.080 |  |
| SF (slope) | | | 0.006 | 0.079 | 0.906 |  |
|  | | | | | |  |
| Model Fit | | | | | |  |
| R^2^ | | | | Marginal | Conditional |  |
|  | | | | 0.780 | 0.776 |  |
| Key: p-values have been calculated using residual approximations.  Confidence intervals calculated using the Wald method  Model equation: SSVEP ~ image type + SF + (1+ image type + SF \| observer) | | | | | |  |

*Supplementary Table 21: Output of the model SSVEP ~ image type + SF + (1+ image type + SF | observer) for the vertical eye channel*


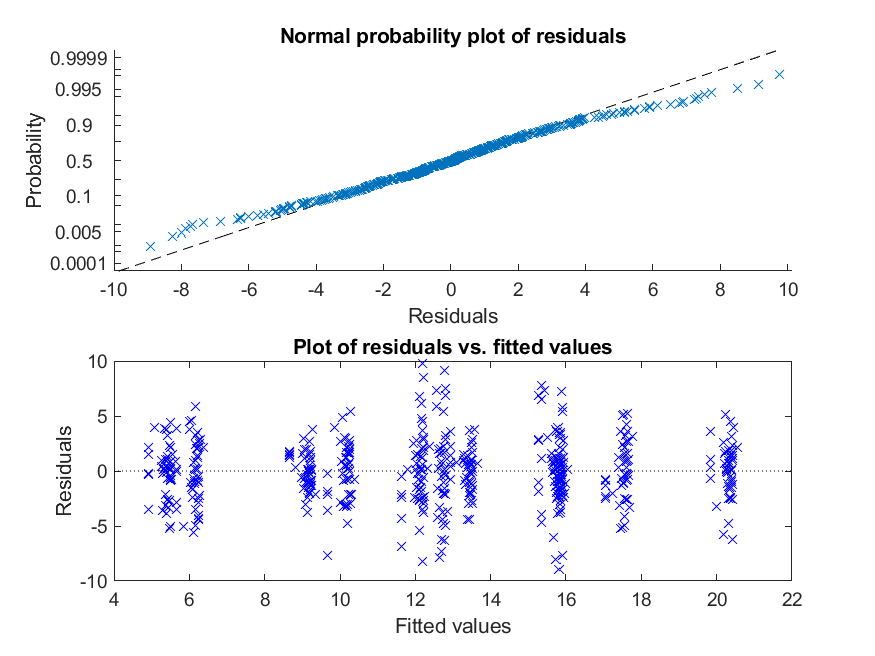


*Supplementary Figure 26: Assumptions of the linear mixed effect model for the model SSVEP ~ PCA1 + PCA2 + PCA3 + (1 | observer) for the vertical eye channel*

| Fixed Effects | | | | | | |
| --- | --- | --- | --- | --- | --- | --- |
|  | Est/Beta | SE | 95% CI | t | p |  |
| Intercept | 12.559 | 1.349 | 9.909 15.208 | 9.3112 | 3.4611e-19 |  |
| PCA1 | 0.082 | 0.057 | -0.028984 0.19362 | 1.4529 | 0.14684 |  |
| PCA2 | 3.729x10-5 | 0.100 | -0.19649 0.19656 | 0.00037264 | 0.9997 |  |
| PCA3 | -0.001 | 0.129 | -0.26288 0.24563 | -0.066628 | 0.9469 |  |
|  |  |  |  |  |  |  |
|  | | | | | |  |
| Random Effects | | | | | |  |
|  | | | Variance | SD | Correlation |  |
| Participant (intercept) | | | 19.847 | 4.455 |  |  |
|  | | | | | |  |
| Model Fit | | | | | |  |
| R^2^ | | | | Marginal | Conditional |  |
|  | | | | 0.717 | 0.715 |  |
| Key: p-values have been calculated using residual approximations.  Confidence intervals calculated using the Wald method  Model equation: SSVEP ~ PCA1 + PCA2 + PCA3 + (1 \| observer) | | | | | |  |

*Supplementary Table 22: Output of the model SSVEP ~ PCA1 + PCA2 + PCA3 + (1 | observer) for the vertical eye channel*

**4) Additional analysis – Does the model predict SSVEP responses?**

The reader may be interested about the relationship between SSVEP responses and the computational model. To this effect, we attempted to assess this, unfortunately the model did not work. Comparisons compared to the null model showed no difference, this can be seen in Supplementary Table 23. Therefore we do not present this in the main paper.

Model equation: SSVEP ~ model + kurtosis + (1|image type) + (1 | observer)

Model building process: SSVEP ~ model + kurtosis + (1|image type) + (1 | observer)

| Model specification | Model name | Nested / simpler model | Fixed effects added |  | | Random Effects | | | | Model Fit | | | | | | LRT Test | | | |
| --- | --- | --- | --- | --- | --- | --- | --- | --- | --- | --- | --- | --- | --- | --- | --- | --- | --- | --- | --- |
|  | | | | |  | | Subjects | | Items | | AIC | BIC | LL | df | df | | Χ^2^ | p |  |
| RE only | Null | - | - |  | | intercepts | | Intercepts | | 2500.90 | | 2518.00 | -1246.40 | 4 |  | |  |  |  |
| Main effects | Main effects 1 | - | Model |  | | intercepts | | Intercepts | | 2499.80 | | 2521.10 | -1244.90 | 5 | 1 | | 3.094 | 0.0786 |  |
|  | Main effects 2 | - | Kurtosis |  | | intercepts | | Intercepts | | 2502.80 | | 2524.20 | -1246.40 | 5 | 1 | | 0.041 | 0.839 |  |
|  | Main effects 3 | - | Model + kurtosis |  | | intercepts | | Intercepts | | 2501.00 | | 2526.60 | -1244.50 | 6 | 2 | | 3.862 | 0.145 |  |

*Supplementary Table 23: Model build table, showing comparison to simpler models. RE = random effects, FE = fixed effects, LRT = simulated likelihood ratio test, AIC = Akaike Information Criterion, BIC = Bayes Information Criterion, LL = log-likelihood, df = degrees of freedom.*

**5) Justification of Sample Size**

There are concerns about underpowered studies in psychology. However, it can be difficult to estimate sample sizes for exploratory studies using linear mixed effects models as an estimate of the coefficients is needed to base simulations on. As we did not in advance know the coefficients on which to base simulations, we did not estimate sample size a priori. However, Brysbaert and Stevens, (2018) make recommendations about the total amount of data, not the number of participants specifically. According to their guidelines they recommend around 1,600 observations for an adequately powered study. In the current study there are 144 observations for each of the 11 observers = 1584 observations, which we argue is adequate for purpose. It has been argued that estimating post-hoc power is not helpful after the fact (Kumle et al., 2021), however, we estimated post-hoc power using the powersim() function in the “simr” package in R (Green and MacLeod, 2016). We used the first analysis to estimate post-hoc power: discomfort ~ 1 + SSVEP + model + kurtosis + (1 | imagetype) + (1 + SSVEP | obs). This was found to have post-hoc power of 82.5%.

**6) Analysis of the Frontal Electrodes**

An additional analysis was conducted to look at the frontal electrodes Fpz, Fp1, Fp2, AFz, AF3, AF4, Fz, F1 and F2 based on the scalp topography.

6.1) Can we predict discomfort from SSVEP and model responses?

A linear mixed effect model was created to predict discomfort from SSVEP from the frontal electrodes from total model response and model kurtosis as fixed effects and observer and image type as random effects. A significant model emerged, see Supplementary Table 24 for the results of the theoretical likelihood ratio test comparing the null model with the full model. Residuals can be seen in Supplementary Figure 27.

| Model specification | Fixed effects | Random effects | df | AIC | BIC | LL | Χ^2^ | df | p |
| --- | --- | --- | --- | --- | --- | --- | --- | --- | --- |
| RE only | - | Subjects (intercepts)  Items  (intercepts) | 6 | 1882.8 | 1908.4 | -935.39 |  |  |  |
| Main effects | Model +  kurtosis | Subjects (intercepts)  Items  (intercepts) | 7 | 1880.4 | 1917 | -930.27 | 10.249 | 3 | 0.02 |

*Supplementary Table 24: Model build table, showing comparison to simpler models. RE = random effects, FE = fixed effects, LRT = simulated likelihood ratio test, AIC = Akaike Information Criterion, BIC = Bayes Information Criterion, LL = log-likelihood, df = degrees of freedom.*


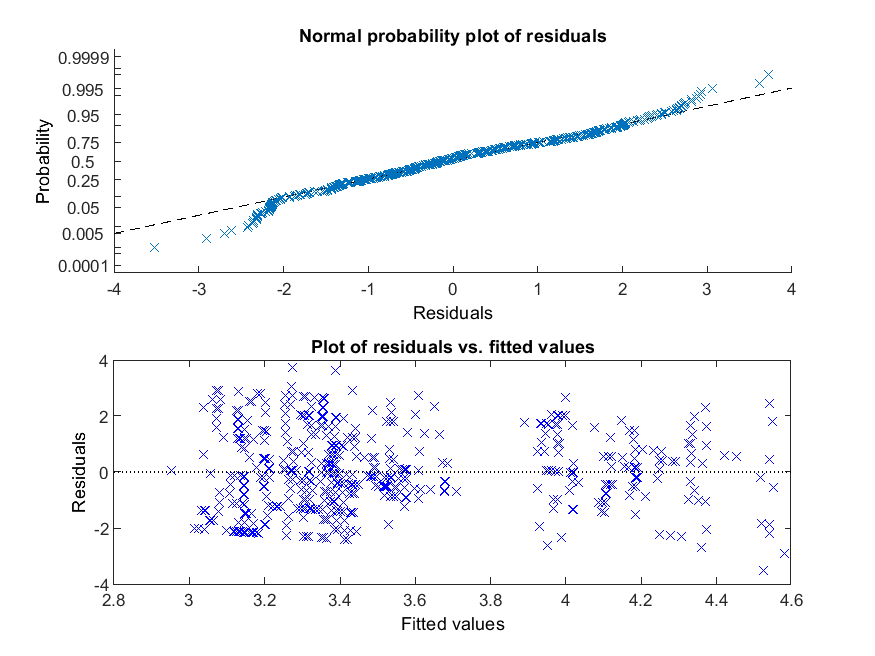


*Supplementary Figure 27: Residuals for the linear mixed effect model for discomfort ~ frontal SSVEP + model + kurtosis + (1 | image type) + (1 + SSVEP | observer)*

Overall the model explained 8% of the variance (adjusted R^2^ = 0.08). The total model response and model kurtosis predicts discomfort judgements, not SSVEP from the frontal areas. The full table of results can be seen in Supplementary Table 25.

| Fixed Effects | | | | | | |
| --- | --- | --- | --- | --- | --- | --- |
|  | Est/Beta | SE | 95% CI | t | P |  |
| Intercept | 3.255 | 0.267 | 2.731 3.779 | 12.211 | 2.36 x10^-30^ |  |
| SSVEP | -0.013 | 0.021 | -0.055 0.028 | -0.630 | 0.529 |  |
| Total Model | 4.81 x10^-7^ | 1.43 x10^-7^ | 1.994x10^-7^ 7.618x10^-7^ | 3.358 | 8.42 x10^-4^ |  |
| Kurtosis | 0.003 | 0.001 | 4.3 x10^-5^ 4.92 x10^-3^ | 2.346 | 0.019 |  |
|  | | | | | |  |
| Random Effects | | | | | |  |
|  | | | Variance | SD | Correlation |  |
| Image type (intercept) | | | 5.275x10^-18^ | 2.967 x10^-9^ |  |  |
| SSVEP (intercept) | | | 1.267x10^-19^ | 3.560 x10^-10^ |  |  |
| SSVEP (slope) | | |  |  | -0.973 |  |
|  | | | | | |  |
| Model Fit | | | | | |  |
| R2 | | | | Marginal | Conditional |  |
|  | | | | 0.08 | 0.08 |  |
| Key: p-values have been calculated using residual approximations.  Confidence intervals calculated using the Wald method  Model equation: Discomfort ~ frontal SSVEP + model + kurtosis + (1 \| image type) + (1 + frontal SSVEP \| observer) | | | | | |  |

*Supplementary Table 25: Output for the model frontal SSVEP ~ model + kurtosis + (1|image type) + (1|observer)*

6.2) Are the smallest SSVEP responses elicited by artworks, and the largest responses elicited by gratings and band-pass filtered noise patterns?

A significant model emerged, when not including image type as a random effect. See Supplementary Table 26 for the results of the theoretical likelihood ratio test comparing the null model with the full model. Residuals can be seen in Supplementary Figure 28.

| Model specification | Fixed effects | Random effects | df | AIC | BIC | LL | Χ^2^ | df | p |
| --- | --- | --- | --- | --- | --- | --- | --- | --- | --- |
| Null | - | Subject (intercept) | 3 | 2250.2 | 2263 | -1122.1 |  |  |  |
| Main effects | Image type | Subject (intercept) | 7 | 2245.2 | 2275.1 | -1115.6 | 13.033 | 4 | 0.01 |

*Supplementary Table 26: Model build table, showing comparison to simpler models. RE = random effects, FE = fixed effects, LRT = simulated likelihood ratio test, AIC = Akaike Information Criterion, BIC = Bayes Information Criterion, LL = log-likelihood, df = degrees of freedom.*


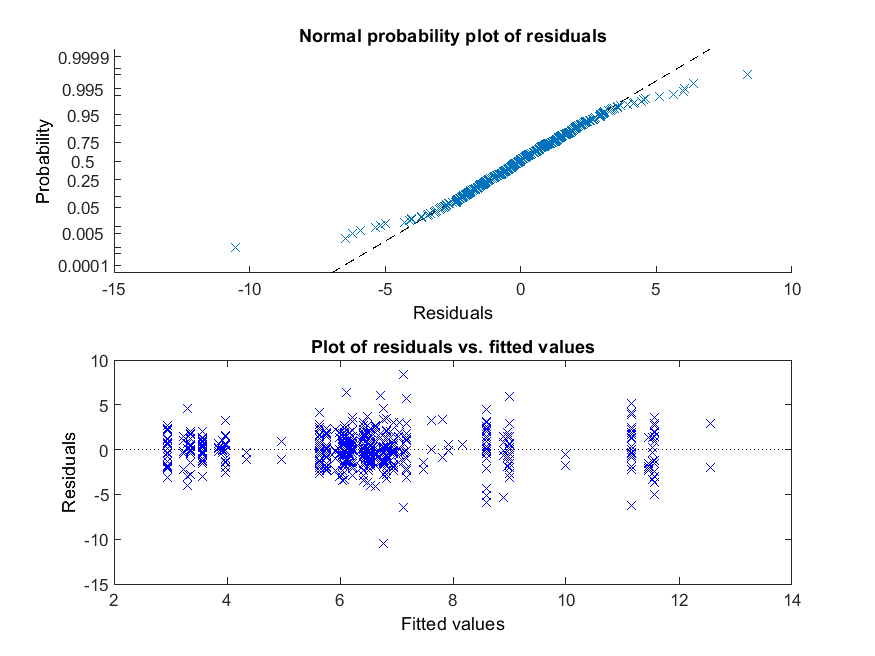


*Supplementary Figure 28: Residuals for the linear mixed effect model frontal SSVEP ~ image type + (1|observer)*

The model predicted 55% of the variance (adjusted R^2^ = 0.55). The work of Bridget Riley predicted the frontal SSVEP judgements, as did the artworks. Artworks reduced the frontal SSVEP response. The work of Bridget Riley increased the frontal SSVEP response. A full table of results can be seen in Supplementary Table 27.

| Fixed Effects | | | | | | |
| --- | --- | --- | --- | --- | --- | --- |
|  | Est/Beta | SE | 95% CI | t | P |  |
| Intercept | 6.745 | 0.658 | 5.451 8.039 | 10.243 | 1.46 x10^-22^ |  |
| Artworks | -0.408 | 0.198 | -0.796 -0.019 | -2.0622 | 0.040 |  |
| Stripes | -0.049 | 0.301 | -0.641 0.543 | -0.162 | 0.872 |  |
| Bump | -0.119 | 0.301 | -0.711 0.473 | -0.396 | 0.693 |  |
| Riley | 0.988 | 0.437 | 0.129 1.847 | 2.260 | 0.024 |  |
|  | | | | | |  |
| Random Effects | | | | | |  |
|  | | | Variance | SD | Correlation |  |
| Participant (intercept) | | | 4.494 | 2.12 |  |  |
|  | | | | | |  |
| Model Fit | | | | | |  |
| R2 | | | | Marginal | Conditional |  |
|  | | | | 0.56 | 0.55 |  |
| Key: p-values have been calculated using residual approximations.  Confidence intervals calculated using the Wald method  Model equation: SSVEP ~ image type + (1 + \|observer) | | | | | |  |

Supplementary Table 27*: Output for the model frontal SSVEP ~ image type + (1|observer)*


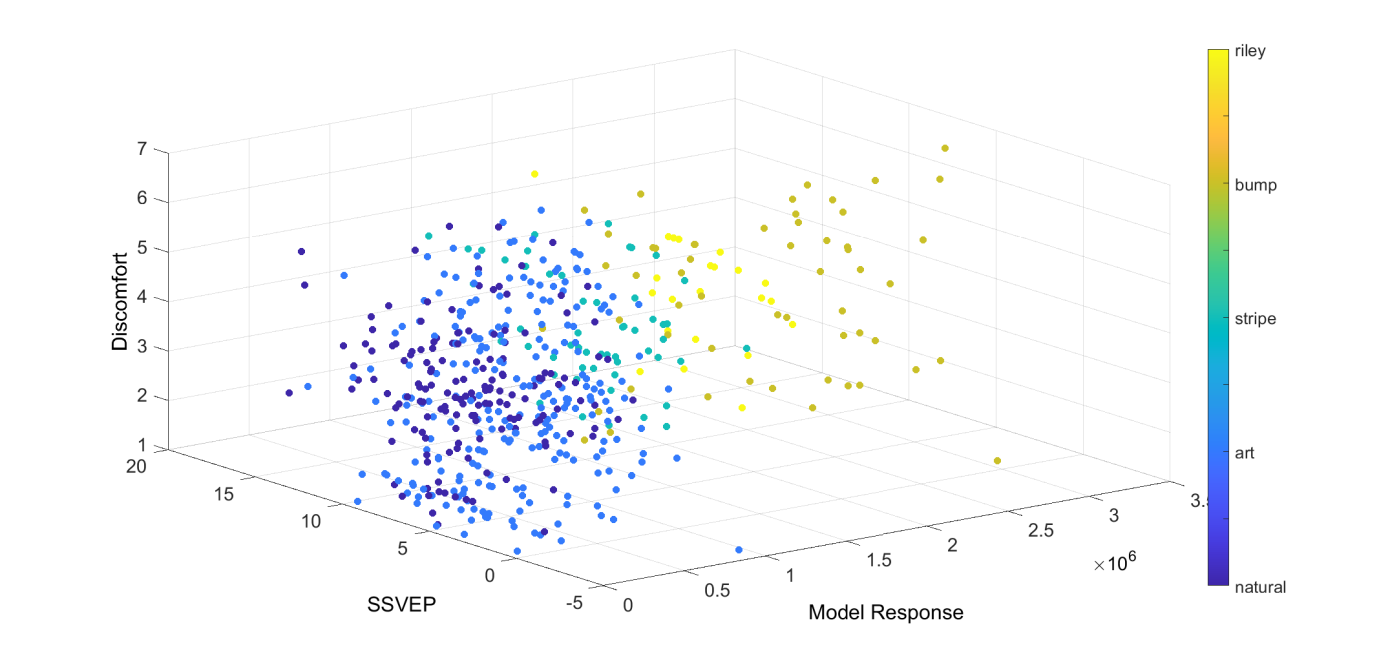


*Supplementary Figure 29: Discomfort judgements predicted by frontal channel SSVEP and total model responses, each colour indicates a different image category.*

6.3) Do mid-range spatial frequencies elicit the greatest discomfort and largest SSVEP responses?

For the SSVEP of the frontal electrodes, there was no significant effect of spatial frequency. The model was not significantly different from null. See Supplementary Table 28 for the results of the theoretical likelihood ratio test comparing the null model with the full model. Residuals can be seen in Supplementary Figure 30.

| Model specification | Fixed effects | Random effects | df | AIC | BIC | LL | Χ^2^ | df | p |
| --- | --- | --- | --- | --- | --- | --- | --- | --- | --- |
| Null | - | Subject (intercept)  Items (intercept and slope) | 8 | 511.07 | 532.67 | -247.53 |  |  |  |
| Main effects | Spatial frequency + image type | Subject (intercept)  Items (intercept and slope) | 10 | 513.66 | 540.66 | -246.83 | 1.405 | 2 | 0.50 |

*Supplementary Table 28: Model build table, showing comparison to simpler models. RE = random effects, FE = fixed effects, LRT = simulated likelihood ratio test, AIC = Akaike Information Criterion, BIC = Bayes Information Criterion, LL = log-likelihood, df = degrees of freedom.*


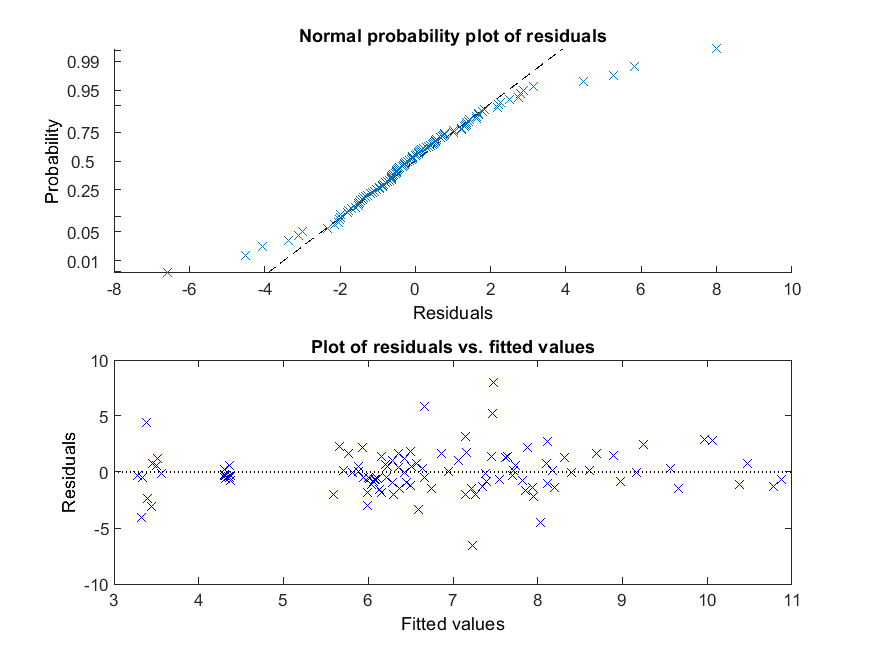


*Supplementary Figure 30: Residuals for the linear mixed effect model for frontal SSVEP ~ image type + spatial frequency + (1 + spatial frequency + image type |observer)*

The model predicted 44% of the variance (adjusted R^2^ = 0.44).

| Fixed Effects | | | | | | | | | | |
| --- | --- | --- | --- | --- | --- | --- | --- | --- | --- | --- |
|  | Est/Beta | SE | 95% CI | | t | | P | |  |  |
| Intercept | 6.189 | 0.664 | 4.873 7.504 | | 9.325 | | 1.72 x10^-15^ | |  |  |
| Image type | -0.071 | 0.389 | -0.842 0.701 | | -0.181 | | 0.857 | |  |  |
| Spatial frequency | 0.169 | 0.143 | -0.115 0.453 | | 1.182 | | 0.240 | |  |  |
|  | | | | | | | | | |  |
| Random Effects | | | | | | | | | |  |
|  | | | | Variance | | SD | | Correlation | |  |
| Participant (intercept) | | | | 2.132 | | 1.46 | |  | |  |
| Image type | | | | 1.000x10^-4^ | | 0.01 | | -1 | |  |
| Spatial frequency | | | | 0.017 | | 0.13 | | 1 | |  |
|  | | | | | | | | | |  |
| Model Fit | | | | | | | | | |  |
| R2 | | | | | | Marginal | | Conditional | |  |
|  | | | | | | 0.45 | | 0.44 | |  |
| Key: p-values have been calculated using residual approximations.  Confidence intervals calculated using the Wald method  Model equation: frontal SSVEP + image type + sf + (1 + spatial frequency + image type \| observer) | | | | | | | | | |  |

Supplementary Table 29 *Output for the model frontal SSVEP ~ image type + spatial frequency + (1 + spatial frequency + image type |observer)*


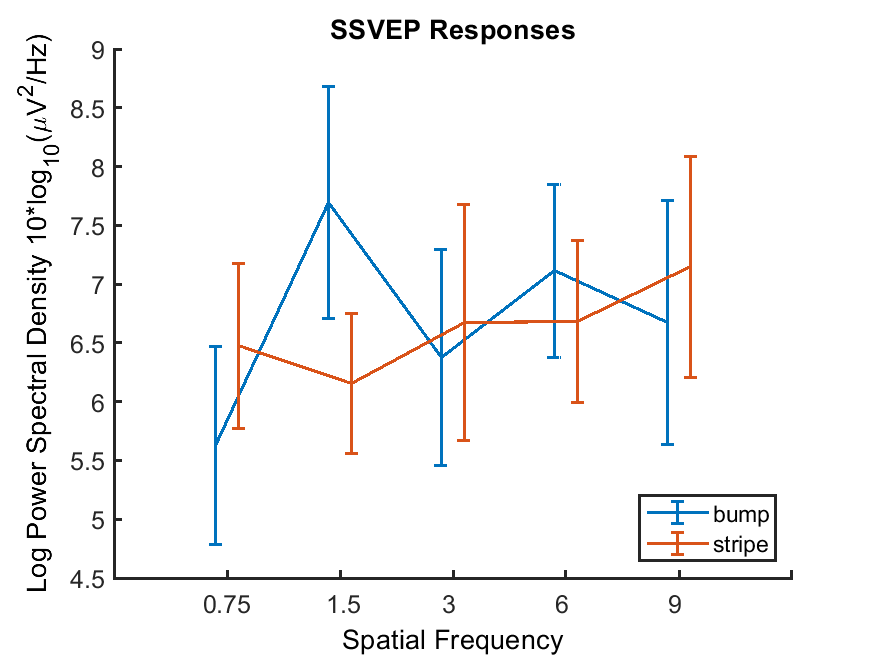


*Supplementary Figure 31: Spatial frequency tuning of SSVEP responses, error bars are ±1SE of the mean.*

6.4) Do low level image statistics predict discomfort judgements and neural responses?

Frontal SSVEP responses were not predicted by PCA1 or PCA2, but they were predicted by PCA3. See Supplementary Table 30 for the results of the theoretical likelihood ratio test comparing the null model with the full model. Residuals can be seen in Supplementary Figure 32.

| Model specification | Fixed effects | Random effects | df | AIC | BIC | LL | Χ^2^ | df | p |
| --- | --- | --- | --- | --- | --- | --- | --- | --- | --- |
| Null | - | Subject (intercept) | 3 | 2250.2 | 2263 | -1122.1 |  |  |  |
| Main effects | PCA1 + PCA2 + PCA3 | Subject (intercept) | 6 | 2247.6 | 2273.2 | -1117.8 | 8.61 | 2 | 0.72 |

*Supplementary Table 30: Model build table, showing comparison to simpler models. RE = random effects, FE = fixed effects, LRT = simulated likelihood ratio test, AIC = Akaike Information Criterion, BIC = Bayes Information Criterion, LL = log-likelihood, df = degrees of freedom.*

| Fixed Effects | | | | | | |
| --- | --- | --- | --- | --- | --- | --- |
|  | Est/Beta | SE | 95% CI | t | P |  |
| Intercept | 6.582 | 0.646 | 5.314 7.850 | 10.196 | 2.16 x10^-22^ |  |
| PCA1 | -0.008 | 0.039 | -0.085 0.068 | -0.219 | 0.826 |  |
| PCA2 | -0.054 | 0.068 | -0.189 0.080 | -0.796 | 0.426 |  |
| PCA3 | -0.250 | 0.088 | -0.424 -0.076 | -2.828 | 0.005 |  |
|  | | | | | |  |
| Random Effects | | | | | |  |
|  | | | Variance | SD | Correlation |  |
| Participant (intercept) | | | 4.494 | 2.12 |  |  |
|  | | | | | |  |
| Model Fit | | | | | |  |
| R2 | | | | Marginal | Conditional |  |
|  | | | | 0.55 | 0.55 |  |
| Key: p-values have been calculated using residual approximations.  Confidence intervals calculated using the Wald method  Model equation: frontal SSVEP ~ PCA1 + PCA2 + PCA3 + (1 \| observer) | | | | | |  |

*Supplementary Table 31: Output of the model SSVEP ~ PCA1 + PCA2 + PCA3 + (1 | observer) for the frontal channels*


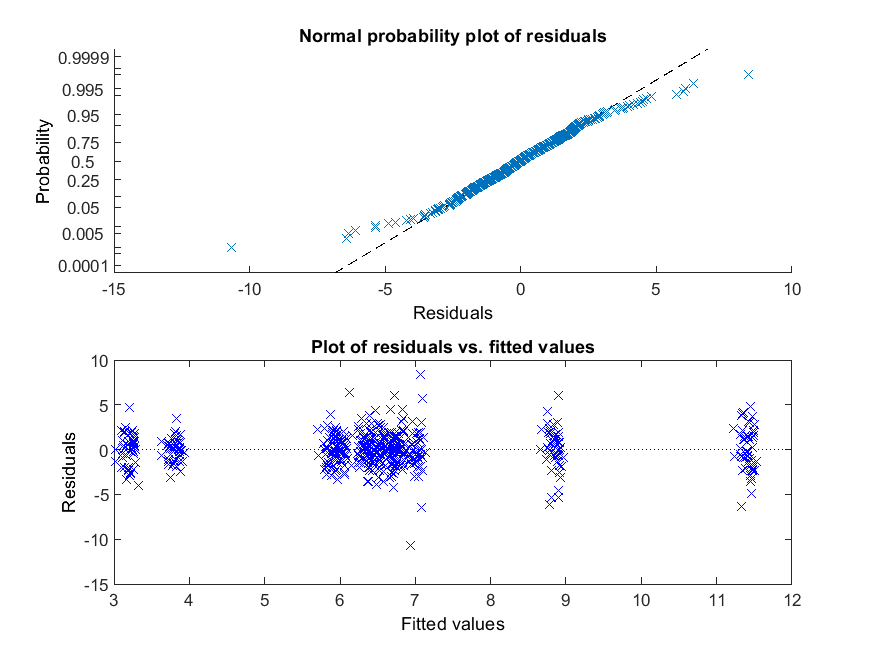


*Supplementary Figure 32: Residuals for the linear mixed effect model SSVEP ~ PCA1 + PCA2 + PCA3 + (1 | observer)*

**7) Analysis of the 10Hz Harmonic**

7.1) Can we predict discomfort from SSVEP and model responses?

A significant model emerged, see Supplementary Table 32 for the results of the theoretical likelihood ratio test comparing the null model with the full model. Residuals can be seen in Supplementary Figure 33.

| Model | Fixed effects | Random effects | df | AIC | BIC | LL | Χ^2^ | df | p |
| --- | --- | --- | --- | --- | --- | --- | --- | --- | --- |
| glme1null | - | Subjects (intercepts)  Items  (intercepts) | 6 | 1882.8 | 1908.4 | -935.39 |  |  |  |
| glme1 | Model +  kurtosis | Subjects (intercepts)  Items  (intercepts) | 9 | 1877.1 | 1915.5 | -929.53 | 11.72 | 3 | 0.008 |

*Supplementary Table 32: Model build table, showing comparison to simpler models. RE = random effects, FE = fixed effects, LRT = simulated likelihood ratio test, AIC = Akaike Information Criterion, BIC = Bayes Information Criterion, LL = log-likelihood, df = degrees of freedom.*


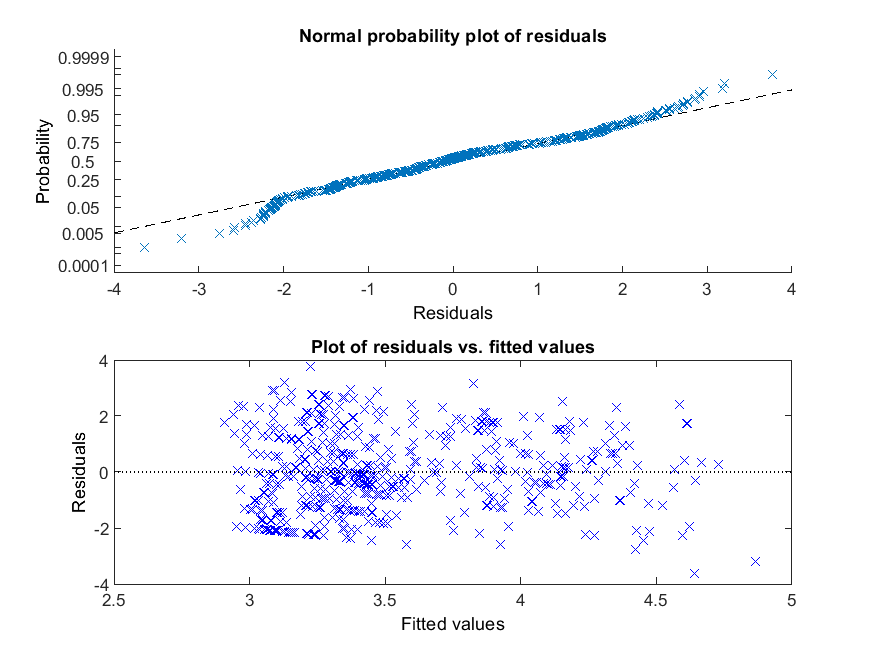


*Supplementary Figure 33: Residuals for the linear mixed effect model for the model 10 Hz SSVEP ~ model + kurtosis + (1|image type) + (1|observer)*

Overall the model explained 8% of the variance (adjusted R^2^ = 0.08). The total model response and model kurtosis predicts discomfort judgements, not SSVEP from the 10Hz harmonic. The full table of results can be seen in Supplementary Table 33.

| Fixed Effects | | | | | | |
| --- | --- | --- | --- | --- | --- | --- |
|  | Est/Beta | SE | 95% CI | t | P |  |
| Intercept | 3.104 | 0.228 | 2.6549 3.552 | 13.585 | 3.16 x10^-36^ |  |
| SSVEP | 0.016 | 0.011 | -0.007 0.038 | 1.3735 | 0.170 |  |
| Total Model | 4.82 x10^-7^ | 1.43 x10^-7^ | 2.02x10^-7^ 7.63x10^-7^ | 3.3778 | 7.85 x10^-4^ |  |
| Kurtosis | 0.003 | 0.001 | 0.0004 0.005 | 2.398 | 0.017 |  |
|  | | | | | |  |
| Random Effects | | | | | |  |
|  | | | Variance | SD | Correlation |  |
| Participant (intercept) | | | 2.657x10^-20^ | 1.63 x10^-10^ |  |  |
| Image type (intercept) | | | 6.452x10^-4^ | 0.254 |  |  |
| SSVEP (slope) | | | 1.96x10^-22^ | 1.40 x10^-11^ | -0.946 |  |
|  | | | | | |  |
| Model Fit | | | | | |  |
| R2 | | | | Marginal | Conditional |  |
|  | | | | 0.09 | 0.08 |  |
| Key: p-values have been calculated using residual approximations.  Confidence intervals calculated using the Wald method  Model equation: Discomfort ~ SSVEP + model + kurtosis + (1 \| image type) + (1 + SSVEP \| observer) | | | | | |  |

*Supplementary Table 33: Output of the model 10 Hz SSVEP ~ model + kurtosis + (1|image type) + (1|observer)*

7.2) Are the smallest SSVEP responses elicited by artworks, and the largest responses elicited by gratings and band-pass filtered noise patterns?

No significant model emerged. See Supplementary Table xxx for the results of the theoretical likelihood ratio test comparing the null model with the full model. Residuals can be seen in Supplementary Figure 34.

| Model | Fixed effects | Random effects | df | AIC | BIC | LL | Χ^2^ | df | p |
| --- | --- | --- | --- | --- | --- | --- | --- | --- | --- |
| glme2null | - | Subject (intercept) | 3 | 2111.5 | 2124.3 | -1052.8 |  |  |  |
| glme2maineffects2 | Image type | Subject (intercept)  Item (intercept) | 21 | 2130.3 | 2220 | -1044.2 | 17.204 | 18 | 0.509 |

*Supplementary Table 34: Model build table, showing comparison to simpler models. RE = random effects, FE = fixed effects, LRT = simulated likelihood ratio test, AIC = Akaike Information Criterion, BIC = Bayes Information Criterion, LL = log-likelihood, df = degrees of freedom.*


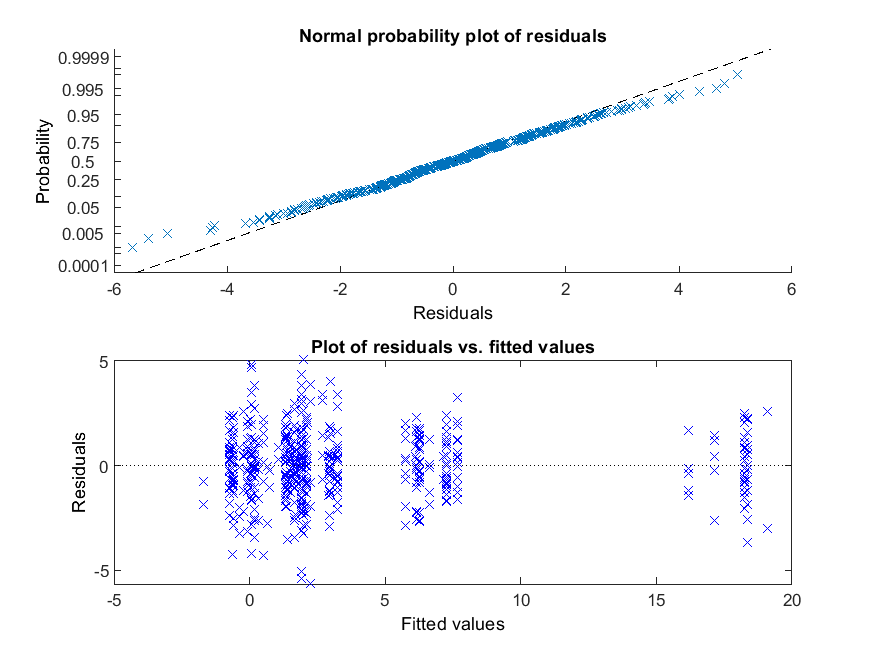


*Supplementary Figure 34: Residuals for the linear mixed effect model 10 Hz SSVEP ~ image type + (1|observer)*

The model predicted 91% of the variance (adjusted R^2^ = 0.91). There was no effect of image type on the SSVEP response at 10Hz harmonic. A full table of results can be seen in Supplementary Table 35.

| Fixed Effects | | | | | | |
| --- | --- | --- | --- | --- | --- | --- |
|  | Est/Beta | SE | 95% CI | t | P |  |
| Intercept | 3.725 | 1.592 | 0.597 6.853 | 2.340 | 0.020 |  |
| Artworks | 0.0505 | 0.207 | -0.357 0.458 | 0.243 | 0.808 |  |
| Stripes | -0.305 | 0.340 | -0.973 0.363 | -0.896 | 0.371 |  |
| Bump | 0.022 | 0.324 | -0.615 0.659 | 0.068 | 0.946 |  |
| Riley | 0.096 | 0.510 | -0.905 1.098 | 0.189 | 0.850 |  |
|  | | | | | |  |
| Random Effects | | | | | |  |
|  | | | Variance | SD | Correlation |  |
| Participant (intercept) | | | 27.668 | 5.26 |  |  |
| Artworks (slope) | | | 0.168 | 0.41 | -0.29 |  |
| Stripes (slope) | | | 0.560 | 0.748 | -0.92 |  |
| Bump (slope) | | | 0.444 | 0.666 | -0.74 |  |
| Riley (slope) | | | 1.357 | 1.165 | 0.05 |  |
|  | | | | | |  |
| Model Fit | | | | | |  |
| R^2^ | | | | Marginal | Conditional |  |
|  | | | | 0.91 | 0.91 |  |
| Key: p-values have been calculated using residual approximations.  Confidence intervals calculated using the Wald method  Model equation: 10Hz SSVEP ~ image type + (1 + image type \| observer) | | | | | |  |

*Supplementary Table 35: Output of the model 10Hz SSVEP ~ image type + (1 + image type | observer)*

7.3) Do mid-range spatial frequencies elicit the greatest discomfort and largest SSVEP responses?

There was no significant effect of spatial frequency on the SSVEP response at the 10Hz harmonic. The model was not significantly different from null. See Supplementary Table xxx for the results of the theoretical likelihood ratio test comparing the null model with the full model. Residuals can be seen in Supplementary Figure 35.

| Model | Fixed effects | Random effects | df | AIC | BIC | LogLik | Χ^2^ | df | p |
| --- | --- | --- | --- | --- | --- | --- | --- | --- | --- |
| Null | - | Subject (intercept)  Items (intercept and slope) | 8 | 490.41 | 512.02 | -237.21 |  |  |  |
| Main effects | Spatial frequency + image type | Subject (intercept)  Items (intercept and slope) | 10 | 492.97 | 519.97 | -236.48 | 1.4495 | 2 | 0.48444 |

*Supplementary Table 56: Model build table, showing comparison to simpler models. RE = random effects, FE = fixed effects, LRT = simulated likelihood ratio test, AIC = Akaike Information Criterion, BIC = Bayes Information Criterion, LL = log-likelihood, df = degrees of freedom.*


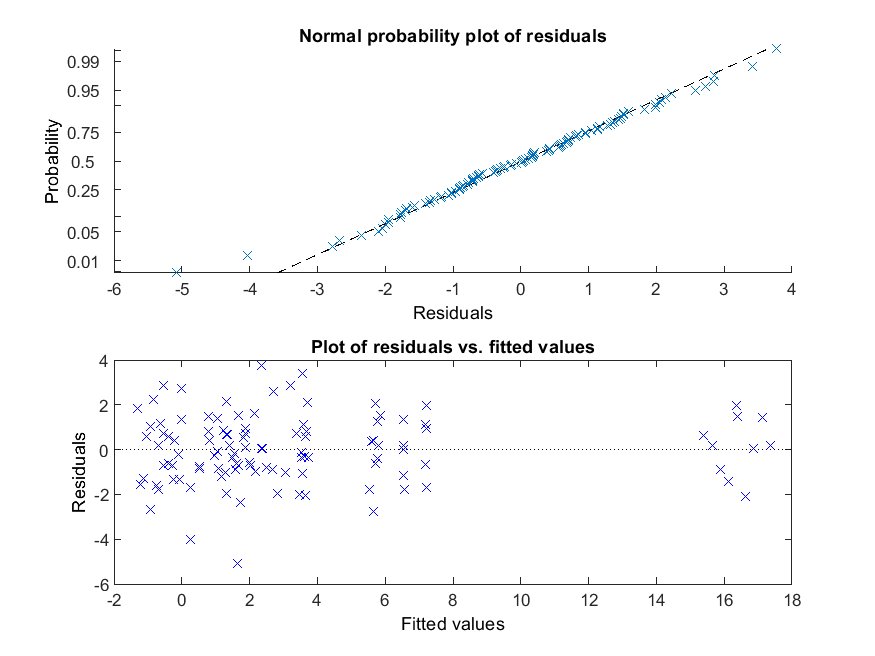


*Supplementary Figure 35: Residuals for the linear mixed effect model 10 Hz SSVEP ~ image type + spatial frequency + (1 + spatial frequency + image type |observer)*

The model predicted 90% of the variance (adjusted R^2^ = 0.90).

| Fixed Effects | | | | | | |
| --- | --- | --- | --- | --- | --- | --- |
|  | Est/Beta | SE | 95% CI | t | P |  |
| Intercept | 3.091 | 1.565 | -0.011 6.193 | 1.976 | 0.051 |  |
| Image type | 0.327 | 0.361 | -0.389 1.043 | 0.905 | 0.368 |  |
| Spatial frequency | 0.110 | 0.130 | -0.14821 0.368 | 0.844 | 0.401 |  |
|  | | | | | |  |
| Random Effects | | | | | |  |
|  | | | Variance | SD | Correlation |  |
| Participant (intercept) | | | 25.221 | 5.022 |  |  |
| Spatial frequency (slope) | | | 0.387 | 0.622 | -0.66 |  |
| Image type (slope) | | | 0.055 | 0.235 | 0.24 |  |
|  | | | | | |  |
| Model Fit | | | | | |  |
| R^2^ | | | | Marginal | Conditional |  |
|  | | | | 0.90 | 0.90 |  |
| Key: p-values have been calculated using residual approximations.  Confidence intervals calculated using the Wald method  Model equation: SSVEP ~ image type + sf + (1 + spatial frequency + image type \| observer) | | | | | |  |

*Supplementary Table 37: Output of the model 10 Hz SSVEP ~ image type + spatial frequency + (1 + spatial frequency + image type |observer)*

7.4) Do low level image statistics predict discomfort judgements and neural responses?

SSVEP responses at the 10Hz harmonic were not predicted by PCA 1 or PCA2, no significant model emerged for the SSVEP response at 10Hz. See Supplementary Table 38 for the results of the theoretical likelihood ratio test comparing the null model with the full model. Residuals can be seen in Supplementary Figure 36.

| Model | Fixed effects | Random effects | df | AIC | BIC | LL | Χ^2^ | df | p |
| --- | --- | --- | --- | --- | --- | --- | --- | --- | --- |
| Null | - | Subject (intercept) | 3 | 2111.5 | 2124.3 | -1052.8 |  |  |  |
| Main effects | PCA1 + PCA2 | Subject (intercept) | 5 | 2115.1 | 2136.4 | -1052.5 | 0.438 | 2 | 0.80 |

*Supplementary Table 38: Model build table, showing comparison to simpler models. RE = random effects, FE = fixed effects, LRT = simulated likelihood ratio test, AIC = Akaike Information Criterion, BIC = Bayes Information Criterion, LL = log-likelihood, df = degrees of freedom.*


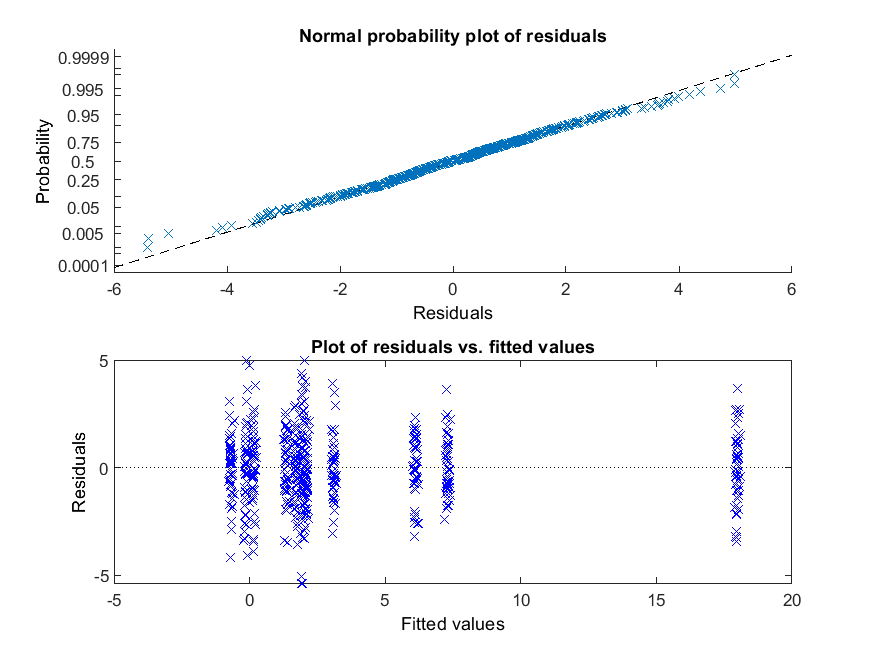


*Supplementary Figure 36: Residuals for the linear mixed effect model 10 Hz SSVEP ~ PCA1 + PCA2 + (1 | observer).*

| Fixed Effects | | | | | | |
| --- | --- | --- | --- | --- | --- | --- |
|  | Est/Beta | SE | 95% CI | t | P |  |
| Intercept | 3.722 | 1.537 | 0.703 6.743 | 2.422 | 0.016 |  |
| PCA1 | 0.001 | 0.034 | -0.065 0.067 | 0.038 | 0.970 |  |
| PCA2 | 0.039 | 0.059 | -0.077 0.155 | 0.661 | 0.509 |  |
|  |  |  |  |  |  |  |
|  | | | | | |  |
| Random Effects | | | | | |  |
|  | | | Variance | SD | Correlation |  |
| Participant (intercept) | | | 25.939 | 5.093 |  |  |
|  | | | | | |  |
| Model Fit | | | | | |  |
| R^2^ | | | | Marginal | Conditional |  |
|  | | | | 0.90 | 0.90 |  |
| Key: p-values have been calculated using residual approximations.  Confidence intervals calculated using the Wald method  Model equation: Discomfort ~ 10 Hz SSVEP +PCA1 + PCA2 + (1 \| observer) | | | | | |  |

*Supplementary Table 39: Output of the model 10 Hz SSVEP ~ PCA1 + PCA2 + (1 | observer)*

**References**

Brysbaert, M., & Stevens, M. (2018). Power analysis and effect size in mixed effects models: A tutorial. Journal of cognition, 1(1).

Green P, MacLeod CJ (2016). “simr: an R package for power analysis of generalised linear mixed models by simulation.” Methods in Ecology and Evolution, 7(4), 493–498. doi:10.1111/2041-210X.12504, https://CRAN.R-project.org/package=simr.

Kumle, L., Võ, M. L. H., & Draschkow, D. (2021). Estimating power in (generalized) linear mixed models: An open introduction and tutorial in R. Behavior research methods, 53(6), 2528-2543.

Schielzeth, H., Dingemanse, N. J., Nakagawa, S., Westneat, D. F., Allegue, H., Teplitsky, C., ... & Araya‐Ajoy, Y. G. (2020). Robustness of linear mixed‐effects models to violations of distributional assumptions. Methods in ecology and evolution, 11(9), 1141-1152.
